# A Multi-Megabase Copy Number Gain Causes Maternal Transmission Ratio Distortion on Mouse Chromosome 2

**DOI:** 10.1371/journal.pgen.1004850

**Published:** 2015-02-13

**Authors:** John P. Didion, Andrew P. Morgan, Amelia M.-F. Clayshulte, Rachel C. Mcmullan, Liran Yadgary, Petko M. Petkov, Timothy A. Bell, Daniel M. Gatti, James J. Crowley, Kunjie Hua, David L. Aylor, Ling Bai, Mark Calaway, Elissa J. Chesler, John E. French, Thomas R. Geiger, Terry J. Gooch, Theodore Garland, Alison H. Harrill, Kent Hunter, Leonard McMillan, Matt Holt, Darla R. Miller, Deborah A. O'Brien, Kenneth Paigen, Wenqi Pan, Lucy B. Rowe, Ginger D. Shaw, Petr Simecek, Patrick F. Sullivan, Karen L Svenson, George M. Weinstock, David W. Threadgill, Daniel Pomp, Gary A. Churchill, Fernando Pardo-Manuel de Villena

**Affiliations:** 1 Department of Genetics, University of North Carolina at Chapel Hill, Chapel Hill, North Carolina, United States of America; 2 Lineberger Comprehensive Cancer Center, University of North Carolina at Chapel Hill, Chapel Hill, North Carolina, United States of America; 3 Carolina Center for Genome Science, University of North Carolina at Chapel Hill, Chapel Hill, North Carolina, United States of America; 4 The Jackson Laboratory, Bar Harbor, Maine, United States of America; 5 Department of Cell Biology and Physiology, University of North Carolina at Chapel Hill, Chapel Hill, North Carolina, United States of America; 6 Department of Biological Sciences, North Carolina State University, Raleigh, North Carolina, United States of America; 7 Laboratory of Cancer Biology and Genetics, National Cancer Institute, National Institutes of Health, Bethesda, Maryland, United States of America; 8 National Toxicology Program, National Institute of Environmental Sciences, NIH, Research Triangle Park, North Carolina, United States of America; 9 Department of Biology, University of California Riverside, Riverside, California, United States of America; 10 Department of Environmental and Occupational Health, University of Arkansas for Medical Sciences, Little Rock, Arkansas, United States of America; 11 Department of Computer Science, University of North Carolina at Chapel Hill, Chapel Hill, North Carolina, United States of America; 12 Jackson Laboratory for Genomic Medicine, Farmington, Connecticut, United States of America; 13 Department of Veterinary Pathobiology and Department of Molecular and Cellular Medicine, Texas A&M University, College Station, Texas, United States of America; University of Wisconsin–Madison, UNITED STATES

## Abstract

Significant departures from expected Mendelian inheritance ratios (transmission ratio distortion, TRD) are frequently observed in both experimental crosses and natural populations. TRD on mouse Chromosome (Chr) 2 has been reported in multiple experimental crosses, including the Collaborative Cross (CC). Among the eight CC founder inbred strains, we found that Chr 2 TRD was exclusive to females that were heterozygous for the WSB/EiJ allele within a 9.3 Mb region (Chr 2 76.9 – 86.2 Mb). A copy number gain of a 127 kb-long DNA segment (designated as responder to drive, *R2d*) emerged as the strongest candidate for the causative allele. We mapped *R2d* sequences to two loci within the candidate interval. *R2d1* is located near the proximal boundary, and contains a single copy of *R2d* in all strains tested. *R2d2* maps to a 900 kb interval, and the number of *R2d* copies varies from zero in classical strains (including the mouse reference genome) to more than 30 in wild-derived strains. Using real-time PCR assays for the copy number, we identified a mutation (*R2d2^WSBdel1^*) that eliminates the majority of the *R2d2^WSB^* copies without apparent alterations of the surrounding WSB/EiJ haplotype. In a three-generation pedigree segregating for *R2d2^WSBdel1^*, the mutation is transmitted to the progeny and Mendelian segregation is restored in females heterozygous for *R2d2^WSBdel1^*, thus providing direct evidence that the copy number gain is causal for maternal TRD. We found that transmission ratios in *R2d2^WSB^* heterozygous females vary between Mendelian segregation and complete distortion depending on the genetic background, and that TRD is under genetic control of unlinked distorter loci. Although the *R2d2^WSB^* transmission ratio was inversely correlated with average litter size, several independent lines of evidence support the contention that female meiotic drive is the cause of the distortion. We discuss the implications and potential applications of this novel meiotic drive system.

## Author Summary

One of the strongest expectations in genetics is that chromosomes segregate randomly during meiosis. However, genetic loci that exhibit transmission ratio distortion (TRD) are sometimes observed in offspring of F1 hybrids. Meiotic drive is a type of non-Mendelian inheritance in which a “selfish” genetic element exploits asymmetric female meiotic cell division to promote its preferential inclusion in ova. We previously reported TRD on Chr 2 in the CC, a mouse recombinant inbred panel with contributions from three *Mus musculus* subspecies. Here we show that maternal TRD consistent with a novel meiotic drive system is caused by a copy number gain. This mutation is similar in size and structure to other known meiotic drive *responders*, such as the knobs of maize. A deletion of most of the copies is sufficient to restore Mendelian segregation, proving that the copy number variant is causative of the observed TRD. In the CC, and also the related DO population, the transmission frequency of the favored allele varies dependent on genetic background, demonstrating that this system is under genetic control. In conclusion, we describe a novel wild-derived meiotic drive locus on mouse Chr 2 that exploits female meiosis asymmetry to violate the Laws of Mendelian inheritance.

## Introduction

Mendel’s Laws provide the theoretical foundation of transmission genetics and explain many of the inheritance patterns of biological traits in sexually reproducing organisms. The Laws state that each gamete receives a random collection of alleles—exactly one per pair of homologous loci—and that gametes unite at random. However, reports of exceptions to Mendelian inheritance date back almost to the rediscovery of Mendel’s Laws, and have been instrumental in elucidating the mechanisms of genetic inheritance [1–4]. Transmission ratio distortion (TRD) is defined as a significant and reproducible violation of the inheritance ratios expected under Mendel’s Laws [1,5–7].

Most observations of TRD are due to selection acting upon the products of meiosis (gamete selection) or fertilization (differential pre- or post-natal survival) [5–8]. The latter is a relatively common occurrence in experimental crosses in many types of organisms including plants and animals [8,9], and is routinely used to classify the essentiality of genes and alleles [9–14]. However, a small but increasing number of observations of TRD can be ascribed to the differential segregation of alleles during meiosis, a process called meiotic drive [1,10–14]. To qualify as such, meiotic drive systems must exhibit three characteristics: 1) asymmetry in the meiotic division(s) with respect to cell fate; 2) functional asymmetry of the meiotic spindle poles; and 3) functional heterozygosity at a locus that mediates attachment of a chromosome or a chromatid to the meiotic spindle [1,15,16]. Meiotic drive is an evolutionary force thought to contribute to karyotypic evolution [15–17] and maintenance of non-essential “B chromosomes” in multiple clades [17,18]. The incidence of meiotic drive is unknown, but given that it is a relatively strong evolutionary force that can lead to the rapid fixation of a selfish allele, it should be rare to observe in action [18–20].

In most plant and animal species meiotic drive is restricted to females, which undergo asymmetric meiosis. At the locus where TRD is observed, an allele that is subject to preferential segregation is termed a responder [19–21]. There are examples in many species of meiotic drive responder alleles that, when in heterozygosity, succeed in being transmitted to the functional product of the asymmetric meiosis more than half of the time (S1 Fig.). Responders in known meiotic drive systems typically involve multi-megabase, highly repetitive and heterochromatic sequences, such as the *D* locus in monkeyflower [11,21], knobs in maize [11,22], homogenously staining regions (HSRs) in wild mice [12,17,22,23] and centromeres and B chromosomes in multiple species [12,17,23,24]. Those systems have mostly proven intractable to molecular characterization, and thus the mechanism(s) by which they gain a segregation advantage are largely unknown. Meiotic drive may be promoted or suppressed by distorter loci (alternately referred to in some publications as effectors, modifiers or drivers).

It is rare for TRD at any single locus to be observed in multiple independent genetic backgrounds. An exception is TRD on mouse Chromosome (Chr) 2, which was reported first in interspecific backcrosses between C57BL/6J (a classical inbred strain, primarily of *Mus musculus domesticus* origin [24–28]) and SPRET/EiJ (a *Mus spretus* wild-derived inbred strain) [25–28]. In offspring from two different (C57BL/6JxSPRET/EiJ)xC57BL/6J backcrosses, the SPRET/EiJ allele was overrepresented across a 40 cM region on Chr 2 [25,28] and a ~140 Mb region on Chr 2 with a maximum transmission frequency of 0.66 [25,29]. TRD in Chr 2 was also reported in an F2 cross between two body weight selection lines, one of which (high body weight; M16i) was derived from the Hsd:ICR outbred stock (also known as CD-1) [29,30]. Additionally, in an advanced intercross between the Hsd:ICR-derived high-running selection line HR8 [30–32] and C57BL/6J, TRD in Chr 2 was present in the primary data but not reported in the corresponding manuscripts [31–35]. And recently, we reported TRD in Chr 2 in the Collaborative Cross (CC) [33–35]. The CC is a mouse recombinant inbred panel derived from eight genetically diverse inbred strains: the classical strains A/J, C57BL/6J, 129S1/SvImJ, NOD/ShiLtJ and NZO/HlLtJ and the wild-derived strains PWK/PhJ (*M*. *m*. *musculus* origin), CAST/EiJ (*M*. *m*. *castaneus*) and WSB/EiJ (*M*. *m*. *domesticus*) [35]. We reported TRD in favor of the WSB/EiJ allele across a ~50 Mb region in the middle of Chr 2 in three largely independent sets of CC lines. In the largest sample, involving 350 genetically independent CC lines, the WSB/EiJ allele was present on 22% of Chr 2 [35,36], a significant over-representation compared to the expected frequency of 12.5% (1/8).

Here we report our extensive genetic characterization of Chr 2 TRD in the CC and in the Diversity Outbred (DO), an outbred population initiated from 144 incompletely inbred CC lines and specifically tailored for high resolution mapping of complex traits [33–36]. Using a combination of classical genetics, whole genome sequence analysis and bioinformatics, we demonstrate conclusively that maternal transmission distortion is caused by a large copy number gain of a 127 kb DNA segment containing a single gene, *Cwc22*. We also provide compelling evidence that meiotic drive is required to explain the TRD in the progeny of heterozygous dams. Finally, we show that there exist several genetically determined levels of TRD controlled by unlinked genetic variation, which, to our knowledge, is unique among meiotic drive systems.

## Results

### Extreme TRD in Chr 2 is present in the DO population

To test whether TRD of the WSB/EiJ allele in Chr 2 is present in the DO, we analyzed 1,175 animals from DO generation 8 (G8) that were genotyped using two related genotyping arrays (MUGA or MegaMUGA, see Materials and Methods). We sampled the genotypes of each individual at 1 Mb intervals along Chr 2 and then computed the overall frequencies of the eight founder alleles at each position. The WSB/EiJ allele was over-represented relative to the other seven founder alleles across a roughly 100 Mb region in the middle of Chr 2 (S2 Fig.). However, there was a striking difference in the level of distortion observed in the CC and the DO, with the WSB/EiJ allele frequency reaching a maximum of 0.22 in the CC compared to 0.55 in the DO. This result indicates that the additional outcrossing in the DO is associated with higher levels of TRD. We conclude that TRD favoring the WSB/EiJ allele is a general feature of crosses in the CC genetic background; however, the level of TRD may vary widely depending on the number of generations of outbreeding.

### TRD is exclusive to heterozygous females

To determine the parental origin of the TRD, we analyzed 5,499 offspring from 18 experimental crosses in which exactly one parent was heterozygous for the WSB/EiJ allele in an interval spanning the region of maximum distortion on Chr 2 (75–90 Mb) [33–35,37,38]. In all cases the heterozygous parent was an F1 hybrid derived either from an intercross between the WSB/EiJ inbred strain and one of eight other inbred strains (the seven founder strains of the CC or PWD/PhJ), or from two CC strains, of which one was homozygous for the WSB/EiJ allele on Chr 2 and the other was homozygous for a non-WSB/EiJ allele. F1 hybrids were mated to either C57BL/6J or FVB/NJ mice, and their progenies were euthanized at birth and genotyped using genetic markers located in the region of maximum distortion. For each cross, we computed the TR of the WSB/EiJ allele and the non-WSB/EiJ allele using the aggregate genotypes across all litters from parents with identical genotypes (Table 1).

**Table 1:** Transmission ratios in the progeny of *R2d2* ^*WSB/notWSB*^ heterozygous F1 hybrid sires and dams.

Cross	Dam	Sire	Informative parent	R2d2^WSB^	R2d2^notWSB^	TR	p
1	C57BL/6J	(WSB/EiJxC57BL/6J)F1	sire	132	136	0.493	8.1x10^–01^
2	C57BL/6J	(C57BL/6JxWSB/EiJ)F1	sire	139	128	0.521	5.0x10^–01^
3	FVB/NJ	(PWK/PhJxWSB/EiJ)F1	sire	263	283	0.482	3.9x10^–01^
4	FVB/NJ	(WSB/EiJxPWK/PhJ)F1	sire	188	171	0.524	3.7x10^–01^
5	FVB/NJ	(CAST/EiJxWSB/EiJ)F1	sire	110	112	0.496	8.9x10^–01^
6	FVB/NJ	(WSB/EiJxCAST/EiJ)F1	sire	98	99	0.498	9.4x10^–01^
7	(WSB/EiJ/CAST/EiJ)F1	C57BL/6J	dam	257	274	0.484	4.6x10^–01^
8	(CAST/EiJxWSB/EiJ)F1	C57BL/6J	dam	248	288	0.463	8.4x10^–02^
9	(PWD/PhJxWSB/EiJ)F1	C57BL/6J	dam	127	142	0.472	8.4x10^–02^
10	(WSB/EiJxPWD/PhJ)F1	C57BL/6J	dam	146	122	0.545	3.6x10^–01^
11	(A/JxWSB/EiJ)F1	FVB/NJ	dam	58	29	0.67	1.4x10^–01^
12	(NODShiLtJ/JxWSB/EiJ)F1	FVB/NJ	dam	135	89	0.6	2.0x10^–03^
13	(129S1/SvImJxWSB/EiJ)F1	FVB/NJ	dam	184	111	0.62	2.0x10^–03^
14	(CC042/GeniUncx CC001/Unc)F1	FVB/NJ	dam	85	38	0.69	2.0x10^–05^
15	(NZO/HILtJxWSB/EiJ)F1	FVB/NJ	dam	130	59	0.69	2.4x10^–07^
16	(CC001/UncxCC039/Unc)F1	FVB/NJ	dam	35	4	0.9	6.9x10^–07^
17	(WSB/EiJxC57BL/6J)F1	C57BL/6J	dam	506	33	0.939	2.9x10^–92^
18	(C57BL/6JxWSB/EiJ)F1	C57BL/6J	dam	512	28	0.948	2.4x10^–96^
Subtotal			sire	930	929	0.500	1.0
Subtotal			dam	2,423	1,217	0.670	7.0x10^–89^

TRs in six paternally segregating crosses (rows 1–6 in Table 1) were as expected under the null hypothesis of Mendelian segregation (range 0.482–0.524, *p* ≥ 0.37). In contrast, the mean TR in maternally segregating crosses (rows 7–18 in Table 1) was 0.666 and deviated significantly from the null hypothesis (*p* = 3.4x10^–89^). We conclude that, in the genetic backgrounds tested, TRD in favor of the WSB/EiJ allele on Chr 2 is restricted to the progeny of heterozygous dams.

The TRs among maternally segregating crosses were significantly different (*p* = 2.4x10^–90^), demonstrating that TRD depends on genetic background (*i*.*e*., TRD is under genetic control). The 12 crosses using F1 hybrid dams can be divided into three classes based on the observed TR (S3 Fig.). F1 hybrid dams derived from crosses between WSB/EiJ and CAST/EiJ or PWD/PhJ showed no distortion (crosses 7–10 in Table 1; aggregate TR = 0.485, 95% CI = 0.46–0.51, *p* = 0.23). Moderate but significant distortion was present in F1 hybrid dams derived from crosses between WSB/EiJ and A/J, 129S1/SvImJ, NZO/HILtJ or NOD/ShiLtJ; and in (CC042/GeniUncxCC001/Unc)F1 hybrid dams (crosses 11–15 in Table 1; aggregate TR = 0.645, 95% CI = 0.61–0.68, *p* = 8.3x10^–19^). Finally, extreme distortion was observed in reciprocal (WSB/EiJxC57BL/6J)F1 hybrid dams and in (CC001/UncxCC039/Unc)F1 hybrid dams (crosses 16–18 in Table 1; aggregate TR = 0.943, 95% CI = 0.93–0.96, *p* = 9.6x10^–193^). We conclude that heterozygosity for the WSB/EiJ allele in the central region of Chr 2 is necessary but not sufficient to observe TRD, because TR was consistent with Mendelian inheritance in some dams that met that criterion.

We also conclude that the grandparental origin of the WSB/EiJ allele has no influence on TRD because the TR levels were not significantly different between three pairs of reciprocal F1 dams (compare crosses 7 and 8, 9 and 10 and 17 and 18 in Table 1; *p* = 0.53, 0.11 and 0.59, respectively).

### TRD maps to a 9.3 Mb interval in the middle of mouse Chr 2

**Figure 1:**
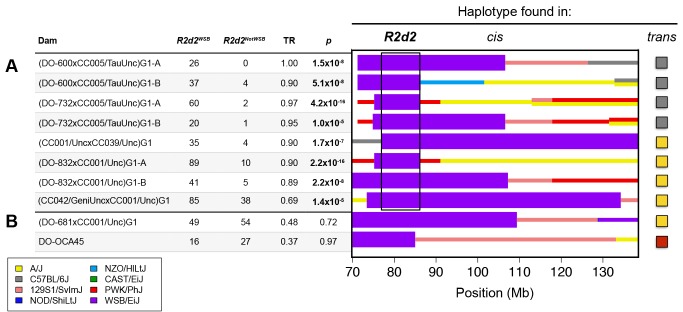
*R2d* maps to a 9.3 Mb candidate interval. CC and DO mice were crossed to generate G1 dams, which were then crossed to FVB/NJ sires to determine the TR in their progeny. Each G1 dam carries a chromosome that is recombinant for the WSB/EiJ haplotype (shown under the heading *cis*) and a non-WSB/EiJ chromosome (the haplotype on the homologue is shown at far right under the heading *trans*). Dams with the same diplotype in the central region of Chr 2 were grouped together to define ten unique diplotypes. The aggregate number of WSB/EiJ and non-WSB/EiJ alleles transmitted by dams of each diplotype are shown for dams A) with TRD and B) without TRD. Significance of TR deviation from Mendelian expectation of 0.5 was computed using one-sided binomial exact test (*p*-value). The contribution from the eight founders of the CC and DO are shown in different colors. Thick purple bars indicate the extent of WSB/EiJ contributions, and thin bars indicate the extent of contributions from all other strains. The black box indicates the boundaries of the *R2d* candidate interval as determined by the region that is WSB/EiJ in all dams with TRD.

To define the boundaries of the locus subject to TRD, we screened 61 CC lines and 378 DO mice that had been genotyped with MegaMUGA for recombinations involving the WSB/EiJ haplotype in the 75–90 Mb interval of Chr 2. We identified five DO females (DO-600, DO-681, DO-732, DO-832 and DO-OCA45) and two CC strains (CC039/Unc and CC042/GeniUnc) that each had at least one informative recombination (Fig. 1). Next, we mated four of the DO females (all except DO-OCA45 that was already heterozygous) and the two CC strains to one of two additional CC lines (CC001/Unc and CC005/TauUnc) that had no contribution from WSB/EiJ on Chr 2, to obtain heterozygous G1 hybrid females. Each hybrid female was genotyped with MegaMUGA and mated to FVB/NJ males (total of 35 crosses; S1 Table).

We found that dams carrying eight of the ten recombinant chromosomes exhibited significant TRD in the Chr 2 interval (TR range 0.69–1.0, *p* ≤ 2.1x10^–5^; Fig. 1 A), but dams carrying two other recombinant chromosomes did not (TR = 0.48 and 0.37, *p* ≥ 0.72; Fig. 1 B). These results are consistent with our conclusion that heterozygosity on Chr 2 is required but not sufficient for TRD; therefore, dams with Mendelian transmission ratios were not used for mapping the locus subject to TRD. Dams with TRD in favor of the WSB/EiJ allele were all heterozygous for a 9.3 Mb interval (the candidate interval; boxed in Fig. 1 A). The proximal boundary of the candidate interval is defined by the recombination found in the CC strain CC039/Unc (*i*.*e*., the most distal SNP inconsistent with a WSB/EiJ haplotype). The distal boundary of the candidate interval is defined by the recombination found in DO-732 and DO-832 females (i.e., the most proximal SNP inconsistent with a WSB/EiJ haplotype). Those SNPs define the maximum boundaries of the locus subject to TRD, Chr 2 76,860,362–86,117,205 (all positions from NCBI/37 unless otherwise noted).

### A multi-megabase copy number gain is associated with TRD on Chr 2

**Figure 2:**
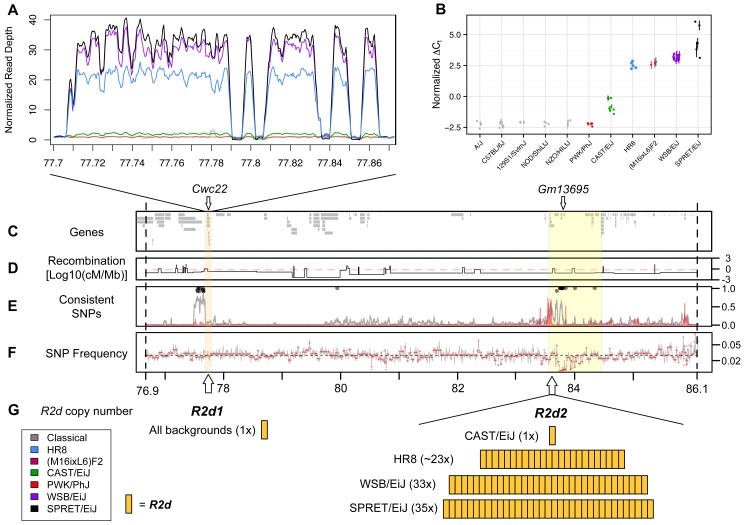
A large copy number gain is present in strains with maternal TRD. A) Read depth in 100 bp windows, normalized by the genome-wide mean read depth for each strain, for *R2d1*, a 158 kb region within the 9.3 Mb candidate interval defined in Fig. 1. *R2d1* includes a single (non-contiguous) copy of *R2d*. Strains are represented by the colors shown in the inset. Inbred strains included under the heading “classical” are A/J, 129S1/SvImJ, C57BL/6JN, NOD/ShiLtJ and NZO/HILtJ. The four large gaps represent LINEs that were inserted in the unique copy found in the reference genome after initial duplication. B) *R2d* copy number estimated by TaqMan assays for *Cwc22*. Normalized cycle threshold (ΔC_t_; see Methods) is proportional to absolute copy number on the log scale. Strains are colored as in panel A. The (M16ixL6)F2 samples shown are known to be homozygous for the M16i allele based on genotypes from the MegaMUGA array. C-F) The yellow boxes highlight the 158 kb region depicted in panel A (*R2d1*) and the 900 kb *R2d2* locus mapping interval. Vertical dashed lines indicate the boundaries of the 9.3 Mb candidate interval. C) Locations of Ensembl genes in the NCBI/37 reference genome within the interval. The locations of the *Cwc22* gene and of seven *Cwc22* pseudogenes (*Gm13695*), are shown. D) Recombination frequency based on Liu et al. (2014), normalized by physical distance (Mb) and log10-transformed. The red line indicates the mean recombination frequency for Chr 2. E) Frequency distribution measured in 1 kb windows of SNPs with shared alleles among the three strains with TRD (WSB/EiJ, SPRET/EiJ and HR8; gray line), and with alleles perfectly consistent between strains with TRD and strains without TRD (A/J, 129S1/SvImJ, C57BL/6JN, NOD/ShiLtJ, NZO/HILt, CAST/EiJ and PWK/PhJ; red line). Lines are smoothed. Black circles indicate windows in which the strains with TRD share an allele for at least 90% of SNPs. F) Frequency distribution of reported SNPs in the candidate interval. G) The location and the number of copies of *R2d* that are present in *R2d1* and *R2d2*.

Among the eight CC founder strains, the candidate interval has 5,018 SNPs, 1,286 small insertions/deletions and 35 structural variants that are private to the WSB/EiJ strain [37–39]. Although this very large number of variants would typically make it difficult to confidently identify and prioritize candidates, one large structural variant has several unique features that made it a strong candidate causative allele for the TRD phenotype. That structural variant is a copy number gain of a 127 kb-long genomic DNA segment (herein referred as *R2d* for responder to drive). In the reference genome, *R2d* is composed of nine non-contiguous sections that, in total, span 158 kb (see *R2d1* locus; Chr 2 77,707,014–77,865,265; Fig. 2 A; S2 Table).

We used the normalized per-base read depth from whole-genome sequence alignments generated by the Sanger Mouse Genomes Project [31,32,37,39] and the HR8 selection line to estimate the number of copies of *R2d* in 18 inbred strains (see Materials and Methods). Similar to C57BL/6J, 15 of the 18 strains, including 5 additional CC founder strains (A/J, 129S1/SvImJ, NOD/ShiLtJ, NZO/HlLtJ and PWK/PhJ) were copy number one (*i*.*e*., a single haploid copy), and CAST/EiJ was copy number two. In contrast, WSB/EiJ had an estimated copy number of 34, and SPRET/EiJ had an estimated copy number of 36, resulting in ~4.4 Mb of additional DNA in those strains (Fig. 2 A). We sequenced 10 individuals from the HR8 selection line (for which Chr 2 TRD was also observed when mated to C57BL/6J [31,32,40]) to a total depth of 125x and aligned the reads to the reference genome. All 10 individuals had evidence of a copy number gain with the same boundaries as in WSB/EiJ and SPRET/EiJ (Fig. 2 A; mean copy number 24.5 +/- 1.4, equating to ~3 Mb of additional DNA).

We used two additional methods to assay the copy number of *R2d*. First, we identified sets of probes on two different genotyping arrays for which the sum hybridization intensity was highly correlated with the copy numbers estimated from sequencing read depth (34 probes in MDA and 3 probes in MegaMUGA; S3 and S4 Tables, respectively). Second, we used real-time quantitative PCR to estimate the *R2d* copy number (Fig. 2 B) using TaqMan assays internal to exons of the single protein-coding gene within *R2d*, *Cwc22* (Fig. 2 C). Using that gene as a proxy for the copy number gain, we found that the copy number estimates from all three methods were highly concordant for the 28 sequenced strains/individuals.

Using the TaqMan assay, we also found that the M16i inbred strain has a high number of copies of *R2d* (Fig. 2 B). We conclude that a large increase (> 20-fold) in *R2d* copy number is found exclusively in strains with TRD (WSB/EiJ, SPRET/EiJ, HR8 and M16i) and that TRD consistently favors the transmission of the allele with the copy number gain.

### The copy number gain maps ~6 MB distal to *R2d1*

**Figure 3:**
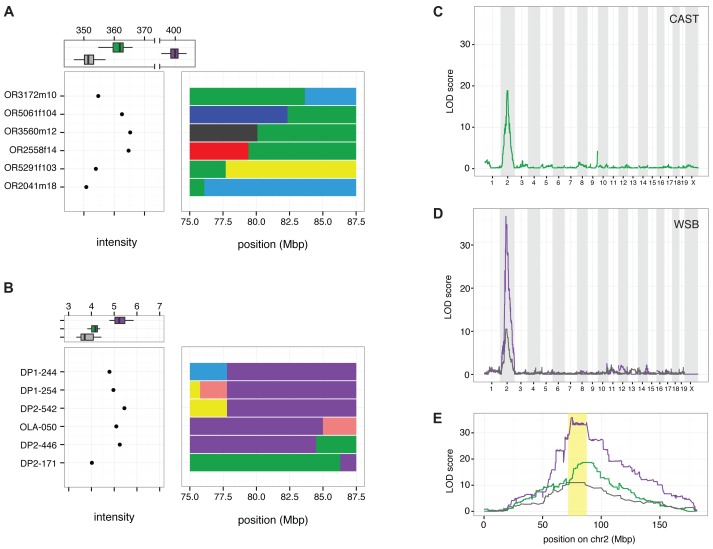
Linkage mapping localizes *R2d2* to a 900 kb region in Chr 2. A) Distribution of sum-intensity for the 34 probes in *R2d* present on the Mouse Diversity Array (MDA) for mice with a non-recombinant CAST/EiJ haplotype (green), a non-recombinant WSB/EiJ haplotype (purple) and non-CAST/EiJ/non-WSB/EiJ haplotypes (grey) is shown at the top of the panel. The sum intensity and recombinant haplotypes in six mice defining the boundaries of copy-number gain in the CAST/EiJ strain are shown below. B) Distribution of sum-intensity across three probes in *R2d* on the MegaMUGA array for mice with non-recombinant CAST/EiJ haplotype (green), a non-recombinant WSB/EiJ haplotype (purple) and non-CAST/EiJ/non-WSB/EiJ haplotypes (grey) is shown at the top of the panel. The sum intensity and recombinant haplotypes in six mice defining the boundaries of copy-number gain in the WSB/EiJ strain are shown below. C) QTL scan for the *R2d2* copy number gain using MDA sum-intensity as the phenotype in 330 CC G2:F_1_ mice. D) QTL scan for the *R2d2* copy number gain using MegaMUGA sum-intensity as the phenotype in 96 (FVB/NJx(WSB/EiJxPWK/PhJ)F1)G2 offspring. E) Superposition of LOD curves from panels (C) and (D) on chromosome 2. The *R2d2* candidate interval is shaded in yellow.

Many structural variants identified from whole-genome sequencing reads have uncertain genomic positions due to the challenge of mapping large variants that are absent from the reference genome. To determine the position of the copy number gain associated with *R2d*, we mapped the WSB/EiJ and CAST/EiJ alleles using segregating populations that have been genotyped at medium (MegaMUGA) or high (Mouse Diversity Array, MDA) density [26,40]. In the CC founder strains, probes located in *R2d* have hybridization intensities correlated with the number of copies estimated from aligned read depth and TaqMan CNV assays (Fig. 2 A, B). The MDA provides robust discrimination between the reference (one copy), CAST/EiJ (two copies) and WSB/EiJ alleles (34 copies; Fig. 3 A). MegaMUGA is able to identify mice carrying the WSB/EiJ allele with little ambiguity (Fig. 3 B). Using the sum intensities of the informative probes as a quantitative trait, we mapped the WSB/EiJ and CAST/EiJ copy number gains in two independent populations and platforms. A genome scan identified a single, broad, highly significant peak on Chr 2 in each population, and those peaks overlap with each other and with the initial candidate interval for TRD (Fig. 3 C-E). We conclude that the copy number gain is closely linked to *R2d1*. This location is consistent with the large copy number gain being the causative allele. Note that both genome scans (Fig. 3 C, D) demonstrate that all the extra *R2d* copies found in WSB/EiJ are located in this interval because no other significant peak is observed in either scan. QTL mapping using TaqMan readout as the phenotype confirmed this result (Fig. 3 D, E).

Analysis of individual mice with recombinant chromosomes in the candidate interval revealed that the copy number gain maps to a 900 kb interval (the *R2d2* locus; Chr 2 83,631,096–84,541,308; Fig. 2; Fig. 3 A, B). Specifically, the CAST/EiJ copy number gain (*R2d2* ^*CAST*^; one additional copy of *R2d*) is located distal to the transition from the CAST/EiJ to the NZO/HILtJ haplotypes found in mice OR3172m10 and OR3172f9 because both mice have low hybridization intensity consistent with a single copy, hence they lack *R2d2* ^*CAST*^ (Fig. 3 A; S4A Fig.). Similarly, the WSB/EiJ copy number gain (*R2d2* ^*WSB*^; 33 additional copies of *R2d*) is located proximal to the transition from the WSB/EiJ to the CAST/EiJ haplotype found on DO mouse DP2–446, because it had high hybridization intensity consistent with the presence of *R2d2* ^*WSB*^ (Fig. 3 B; S4B Fig.). These results demonstrate that *R2d2* is not located immediately adjacent to *R2d1* but approximately 6 Mb distal to it. The distal location of the copy number gain is confirmed by the analysis of the sum intensity of the three MegaMUGA probes that track *R2d* in two backcrosses involving the SPRET/EiJ inbred strain [26,41] (S4C Fig.).

### Loss of *R2d* copies at *R2d2* restores Mendelian transmission of Chr 2

**Figure 4:**
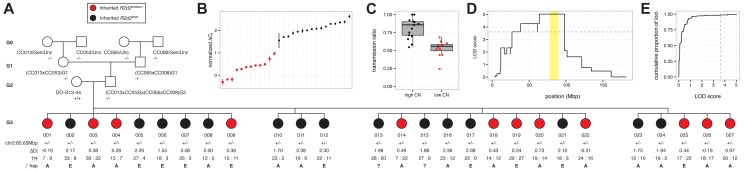
Mapping the causal locus for maternal TRD in a family segregating for a copy-number variant at *R2d2*. A) Pedigree of DO-G13–44xCC cross. Female DO-G13–44, mother of the G3 dams phenotyped for TR, is segregating for a copy-number variant at *R2d2*. G3 dams inheriting the maternal WSB/EiJ haplotype associated with the high-copy allele (*R2d2* ^*WSB*^) are colored black; those inheriting the WSB/EiJ haplotype associated with the low-copy allele (*R2d2* ^*WSBdel1*^) are colored red. Genotypes at marker chr2:85.65Mbp is denoted -/- (homozygous non-WSB), +/- (heterozygous WSB/EiJ) or +/+ (homozygous WSB/EiJ). ΔC_t_, normalized cycle threshold by TaqMan qPCR assay; TR, transmission ratio, denoted as count of progeny inheriting a WSB/EiJ allele: count of progeny not inheriting a WSB allele; the paternal haplotype at chr2:83.6 Mb as determined by genotypes from the MegaMUGA array using the standard CC abbreviations is shown, A = A/J, E = NZO/HILtJ, ? = haplotype unknown. B) Distribution of ΔC_t_ values among 27 G3 dams. Points are colored as in panel A. C) TR among 27 G3 dams partitioned according to copy-number (CN) haplotype at *R2d2*. Points are colored as in panel A. D) QTL scan for TRD, treated as a binary phenotype, in 25 G3 dams genotyped with MegaMUGA. Only the maternal signal from Chr 2 is shown. Grey dashed line indicates threshold for significance at *α* = 0.01 obtained by unrestricted permutation. Candidate interval for *R2d* is shaded yellow. E) Empirical cumulative distribution of both maternal and paternal LOD scores genome-wide, with *α* = 0.01 significance threshold indicated by grey dashed line.

We used the TaqMan assay to confirm *R2d* copy number in all heterozygous females tested for TRD (S1 Table; S5 Fig.). We identified a dam (DO-G13–44) that was homozygous for the WSB/EiJ haplotype across the entire candidate interval but produced offspring that were segregating for the copy number gain (Fig. 4 A). This was confirmed by estimating *R2d* copy number in each of 27 G3 females and 16 G4 progeny that were heterozygous for a WSB/EiJ haplotype (Fig. 4 B; S5 Fig.). We determined the TR in 825 progeny of G3 dams mated to FVB/NJ sires. The TRs among the 27 G3 dams were significantly different (*p* = 4.9x10^–12^). In the progeny of the 15 G3 dams with high copy number there was significant TRD in favor of the WSB/EiJ allele (TR = 0.78, *p* = 2x10^–30^; Fig. 4 C). In contrast, we found absence of TRD in the 12 G3 dams that inherited the low-copy allele (TR = 0.53, *p* = 0.234). A genome scan for TRD as a binary trait demonstrated that presence or absence of TRD in this pedigree maps uniquely to the candidate interval (Fig. 4 D, E).

We were also able to estimate that G3 dams with the low-copy allele had a copy number of ~11. We conclude that the loss of ~22 copies of *R2d* was sufficient to rescue Mendelian transmission, thus demonstrating that the copy number gain is causative of TRD.

### Meiotic drive is the most likely cause of maternal TRD at *R2d2*

**Figure 5:**
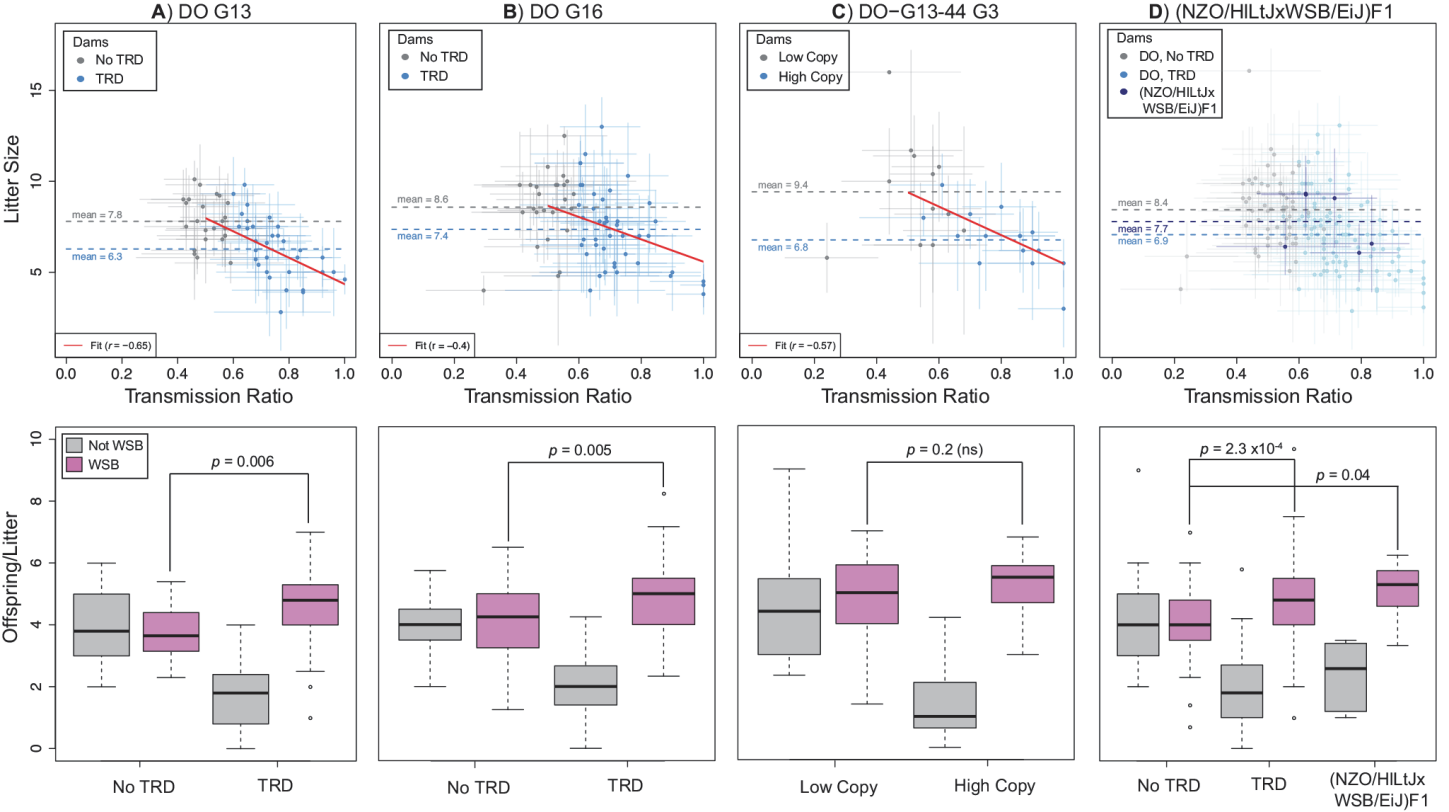
TRD at *R2d2* requires the combined action of meiotic drive and embryonic lethality. Relationship between maternal TR and average litter size (top panels) and average number of offspring inheriting alternative alleles at R2d2 (bottom panels) for A) DO G13 dams, B) DO G16 dams, C) G3 dams in the D0-G13–44 pedigree and D) (NZO/HILtJxWSB/EiJ)F1 dams. Top panels: gray circles are dams without TRD (A, B, D) or having the low-copy allele (C); blue circles are dams with TRD (A, B, D) or having the high-copy allele (C). For each point, bars show standard error for TR (horizontal) and average litter size (vertical). Dotted lines show mean litter sizes for each type of female. Red line shows a linear fit to TR and average litter size. Bottom panels: left and right pairs of boxplots show average number of offspring per litter in females without and with TRD (A, B, D) or having the low- and high-copy allele (C) that inherit a WSB/EiJ (purple) or non-WSB/EiJ (gray) allele. Females with a mutant *R2d2* ^*WSB*^ allele are excluded. Note that there are significantly more WSB/EiJ offspring of dams with TRD in F1 hybrid dams than in DO without TRD.

The results presented above demonstrate that TRD at *R2d2* is only observed in the progeny of heterozygous dams. This restricts the plausible causes of TRD to meiotic drive, genotype-dependent embryonic lethality (including genotype-dependent competition between embryos) or a combination of both. To identify the cause of TRD, we first determined whether TR levels (S6 Fig.; S1 Table) were correlated with litter size in 127 DO dams (these 56 DO-G13 and 71 DO-G16 females are a random sample from an outbred population). We observed a strong inverse correlation between average litter size and TR at *R2d2* (*r* = -0.65, *p* = 7.2x10^–8^ and *r* = -0.40, *p* = 5x10^–4^ in the DO-G13 and DO-G16 dams, respectively; Fig. 5 A, B). We conclude that the presence and the strength of TRD are significantly associated with reduced litter sizes and thus with some type of embryonic lethality. We determined the relationship between TRD and litter size under the assumption of TRD caused exclusively by embryonic lethality [40,41] (S7 Fig.). Under this scenario, in both the DO-G13 and DO-G16 samples the observed average litter size is significantly greater than predicted based on TR (*p* = 0.021 and 6.0x10^–5^ for DO-G13 and DO-G16 dams, respectively; S7 Fig.). We conclude that embryonic death alone could only account for a fraction of the “missing” progeny inheriting a non-WSB/EiJ (*R2d2* ^*NotWSB*^) allele. We determined directly the levels of embryonic lethality in DO-G13 dams at mid-gestation (see Materials and Methods). We observed that dams with TRD had slightly, but not significantly, higher numbers of resorbed embryos present *in utero* than did dams with Mendelian segregation (1.3 ± 1.5 and 1.1 ± 1.2 resorbed embryos, respectively, *p* = 0.66; N = 29 and 19 dams, respectively; S8 Fig.). We conclude that embryonic lethality alone is insufficient to explain TRD at *R2d2*.

Although embryonic lethality can change the proportion of progeny inheriting alternative alleles at *R2d2*, only meiotic drive can lead to an increase in the absolute number of progeny inheriting the *R2d2* ^*WSB*^ allele per litter in dams with TRD compared to dams with Mendelian segregation. To test whether meiotic drive was responsible for TRD, we determined the average absolute number of offspring per litter that inherited the *R2d2* ^*WSB*^ and *R2d2* ^*NotWSB*^ alleles in the progenies of the DO-G13 and DO-G16 DO dams with either TRD or Mendelian segregation. In dams with Mendelian segregation, the average numbers of offspring per litter that inherited either allele were not different (3.80 *R2d2* ^*WSB*^ versus 3.96 *R2d2* ^*NotWSB*^, *p* = 0.73 in DO-G13 dams; 4.13 *R2d2* ^*WSB*^ versus 4.03 *R2d2* ^*NotWS*^, *p* = 0.29 in DO-G16 dams; Fig. 5 A, B). In contrast, in the progenies of dams with TRD the average number of offspring per litter that inherited the *R2d2* ^*WSB*^ allele (4.51 and 4.89 in the DO-G13 and DO-G16 dams, respectively) was significantly greater than the absolute number of either allele in the offspring of dams without distortion (*p* = 0.006 and 0.049 for the *R2d2* ^*WSB*^ and *R2d2* ^*NotWSB*^ alleles in DO-G13; *p* = 0.005 and 4x10^–4^ for the *R2d2* ^*WSB*^ and *R2d2* ^*NotWSB*^ alleles in DO-G16; Fig. 5 A, B). The same result holds true for live embryos at mid-gestation: the average numbers of offspring that inherited *R2d2* ^*WSB*^ and *R2d2* ^*NotWSB*^ alleles were 5.0 ± 2.2 and 1.6 ± 1.8 for dams with TRD versus 4.3 ± 1.6 and 3.4 ± 1.8 for dams without TRD. Based on the consistent and significant excess average absolute number of *R2d2* ^*WSB*^ alleles in the litters of dams with TRD, we conclude again that meiotic drive is required to explain TRD at *R2d2*.

Further support for meiotic drive was provided by the analysis of the DO-G13–44 pedigree (Fig. 5 C) and crosses between (NZO/HILtJxWSB/EiJ)F1 dams and FVB/NJ sires (cross 15 in Table 1; Fig. 5 D). The average litter size of DO-G13–44 G3 dams inheriting the mutant *R2d2* ^*WSB*^ allele (*R2d2* ^*WSBdel1*^) was larger than in dams inheriting the standard *R2d2* ^*WSB*^ allele (9.4 ± 2.9 and 6.8 ± 1.6, respectively), but the observed average litter size in dams with TRD is significantly greater than predicted based on TR (*p* = 0.02; S7 Fig.). Similarly, in the (NZO/HILtJxWSB/EiJ)F1 crosses the average litter size (7.7 ± 2.4; Fig. 5 D) was comparable to DO-G13 and DO-G16 dams without TRD, and was greater than predicted based on TR (*p =* 0.09; Fig. 5). There was little direct evidence of embryonic lethality at mid-gestation (1.8 ± 1.6 and 0.4 ± 0.5 resorbed embryos, respectively; S8 Fig.). Furthermore, DO-G13–44 G3 dams with different *R2d2* alleles differed significantly in the average absolute number of offspring per litter inheriting the *R2d2* ^*WSB*^ allele (in dams with TRD) compared to the *R2d2* ^*WSBdel1*^ allele (in dams with Mendelian segregation; 5.3 ± 2.0 and 4.64 ± 2.4, respectively, *p* = 0.07; Fig. 5 C). Similar results are observed when comparing the absolute number of offspring per litter that inherited the *R2d2* ^*WSB*^ allele in the (NZO/HILtJxWSB/EiJ)F1 crosses to the DO dams without TRD (5.1 ± 1.0 and 4.1 ± 1.1, respectively, *p* = 0.03; Fig. 5 D). In summary, all data from four independent experimental populations were consistent with an explanation of Chr 2 TRD that requires the joint presence meiotic drive and low-level embryonic lethality.

## Discussion

### A large copy number variant causes maternal TRD and reduces the average litter size in heterozygous dams

After demonstrating that TRD occurs only through the germline of F1 female mice, we were faced with two major obstacles in our efforts to map the causative locus. First, although heterozygosity for the WSB/EiJ allele is required, it is not sufficient for meiotic drive (Table 1; S1 Table). Therefore, we initially mapped the responder by determining the minimum region of overlap for the WSB/EiJ haplotype only in dams with TRD (Fig. 1). This yielded a 9.3 Mb candidate interval. Second, the candidate interval spans a recombination-cold region [37,40,42], and the frequency of recombination is three-fold lower than expected in the CC (Fig. 2 D). Although this likely contributes to the overall deficit in recombinant chromosomes (none observed versus an expected 23 in the 378 DO females and 4 in 61 CC lines), the complete lack of recombinants involving the WSB/EiJ haplotype is striking, and, for the purposes of this study, a major impediment to the precise mapping the responder.

Within the candidate interval, a single variant (*R2d2*) stands out as the most likely cause of TRD. *R2d2* consists of one or more copies of a 127 kb sequence (*R2d*). High copy number (≥ 24) is present in all four strains with reported TRD and low copy number (≤ 2) is present in all eight strains without TRD (Fig. 2 A, C). The expansion in copy number leads to an increase of at least 3 Mb in DNA content within the allele favored by maternal TRD. Among CC founders, only WSB/EiJ has a high copy number allele.

As the reference genome is based on a single classical inbred strain, C57BL/6J, copy number gains in other strains or wild mice may be located in a different physical location. Fortunately, the presence of a third allele in CAST/EiJ (which exhibited a twofold enrichment of sequencing reads) combined with the fact that recombinations involving the CAST/EiJ haplotype are not suppressed within the 9.3 Mb candidate interval, enabled us to map the physical location of *R2d2* to a 900 kb region located 6 Mb distal to *R2d1*, the locus where the sequencing reads mapped in the reference genome (Fig. 3). Importantly, the mapping of *R2d2* was enabled by the availability of deep sequence data for each of the strains used in our experiments [25–27,37,42,43 and this study] and by combining the results of experiments completed 20 years apart [25–27,43,44].

We determined the number and spatial distribution of SNPs in the 9.3 Mb candidate interval that partition the ten inbred strains with whole genome sequence in a pattern consistent with the TRD phenotype (three strains with TRD: WSB/EiJ, SPRET/EiJ and HR8; and seven strains without TRD: A/J, C57BL6/J, 129S1/SvImJ, NOD/ShiLtJ, NZO/HILtJ, CAST/EiJ and PWK/PhJ). Compared to a genome-wide mean of 1 consistent SNP every ~3.2 kb, within the 900 kb region where we mapped *R2d2* there was a mean of 1 consistent SNP every 883 bp (*p* < 1.0x10^–4^, one-sided Student’s *t*-test; Fig. 2 E). This reduction in diversity is not due to undercalling of SNPs in the *R2d2* candidate interval (Fig. 2 F). The fact that consistent SNPs are rare in most of the genome but are common within the 900 Kb region in which *R2d2* maps supports the hypothesis that *R2d2* is the causative allele for TRD.

Most importantly, we identified a DO female (DO-G13–44) that was homozygous for the WSB/EiJ haplotype across the entire *R2d* candidate interval but was heterozygous for *R2d2* alleles with different copy numbers (Fig. 4). We generated a three-generation pedigree and analyzed the *R2d* copy number, the Chr 2 haplotype and TR in the progeny of heterozygous dams with different copy numbers. This analysis revealed perfect correlations between the inheritance of *R2d2* ^*WSBdel1*^ and complete absence of TRD in favor of the WSB/EiJ allele, and between the inheritance of *R2d2* ^*WSB*^ and presence of TRD. This experiment demonstrates that the reduction in copy number from 33 to 11 is sufficient to restore Mendelian segregation, and that *R2d2* is the causative allele for maternal TRD.

Further evidence that TRD requires an *R2d2* allele with copy number of above 11 is provided by the NU/J inbred strain. This strain has intermediate copy number (7, estimated by TaqMan) but no TRD in the progeny of (NU/JxC57BL/6J)F1 female hybrids (0.55, *p* = 0.55; S12 Fig.).

### Gene content and sequence composition of *R2d*

The presence of *R2d* sequences at two distinct locations (Fig. 2 G) indicates an initial duplication of this segment in the ancestor of CAST/Eij, WSB/EiJ, SPRET/EiJ and Hsd:ICR. *R2d s*pans a highly expressed protein coding gene (*Cwc22*; Fig. 2 C) that is implicated in RNA splicing [38,44], a predicted gene of unknown function that overlaps with the last exon of *Cwc22* (*Gm13727*) and a pseudogene (*Gm13726*). DNA copy number variation for *Cwc22* has been described previously [38,45]. *Cwc22* is highly expressed in mouse oocytes and fertilized eggs [45,46]. The *Cwc22* gene is a known eQTL in mouse: allele-specific RNA-seq of brain tissue from reciprocal crosses between WSB/EiJ, PWK/PhJ and CAST/EiJ showed extreme differential expression, with the WSB/EiJ allele more highly expressed than the other two [46,47].

Apart from its size and repetitive nature, an important feature of the *R2d2* locus is its remarkable uniformity between three divergent genetic backgrounds that are separated by ~1 million years of evolution: WSB/EiJ, SPRET/EiJ and HR8 [47–49]. For example in WSB/EiJ and SPRET/EiJ the genome-wide mean is 1 SNP every ~60 bp [37] and the mean SNP frequency within *R2d* is significantly reduced to 1 SNP every 1,342 bp (*t-test*, *p* = 3.9x10^–58^). Further analysis will be required to determine the respective ages of the duplication and the copy number change(s), and whether interspecific introgression [48–51] is required to explain the unlikely degree of sequence conservation between *M*. *m*. *domesticus* and *M*. *spretus*.

We note that, while unlikely given the results of our QTL mapping (Fig. 3), it is possible that there have been additional duplication events that have also inserted *R2d* in other chromosomes. Additionally, the causal allele may incorporate additional DNA sequences, including some that may be absent in the reference genome (similar to the origin of the sequence on maize chromosome Ab10 that causes meiotic drive in that species). If that is the case, the causal allele may be much larger than 4.4 Mb. For example, HSR alleles as large as 200 Mb have been described [50–52].

### How do meiotic drive and embryonic lethality contribute to TRD at *R2d*?

A second focus of our study was to discriminate among the many mechanisms [29,52] that could give rise to TRD at *R2d2*, and to rule out as many as possible. First, the fact that TRD is only observed through the maternal germline rules out both spermatogenesis-mediated processes and sperm competition. Second, the presence of TRD at birth rules out differential survival of offspring. Third, the fact that distortion was independent of the maternal granddam precludes cytoplasmic effects. The remaining plausible explanations are differential fertilization based on the oocyte genotype, embryonic lethality and/or meiotic drive. The first two mechanisms should reduce the average litter size proportionally to TR (black line in S7 Fig.), while the average absolute number of offspring inheriting the favored genotype (*R2d2* ^*WSB*^) per litter remains constant. The number of resorbed embryos observed in pregnant females could distinguish the two mechanisms because it should be greater in the second than in the first scenario. In contrast, if meiotic drive is solely responsible for TRD then the following should be true: 1) average litter size is independent of TRD, 2) the average absolute number of offspring inheriting the favored genotype (*R2d2* ^*WSB*^) per litter is higher in dams with TRD than in dams with Mendelian segregation, and 3) the level of embryonic lethality is independent of the presence and level of distortion. The data shown in the Results section are most consistent with the combined action of embryonic lethality and meiotic drive. Specifically, meiotic drive is required to explain both the fact that the observed average litter size in the DO-G13 and DO-G16 dams, in the DO-G13–44 pedigree and in the (NZO/HILtJxWSB/EiJ)F1 dams is greater than predicted based on TR (S7 Fig.), and that the average absolute number of offspring inheriting the *R2d2* ^*WSB*^ genotype per litter is greater in dams with TRD (Fig. 5). Note that some *p*-values in comparisons involving (NZO/HILtJxWSB/EiJ)F1 crosses failed to reach statistical significance due to the small sample size, but the trends were always consistent with those in DO dams with TRD.

An alternative explanation that does not involve meiotic drive would require the combined presence of increased ovulation in dams with TRD and pre- or post-implantation genotype-dependent competition between embryos favoring the allele with the high copy number at *R2d2*. Genotyping at *R2d2* and re-analysis of 159 F2 females from the M16ixL6 intercross [29] confirms an overdominant effect of the *R2d2* genotype in the number of live and dead embryos at day 16 of gestation, as predicted under the meiotic drive and embryo competition scenarios, but shows no effect of the *R2d2* locus on ovulation rates (S9 Fig.). This result is not due to a lack of power, as we have 80% power (at a = 0.5) to detect a difference in the mean ovulation rate du to an effect of the *R2d2* genotype and QTLs for ovulation rate were identified in the original study [29,53–55]. In summary, the effect of the *R2d2* genotype on reproductive phenotypes is most consistent with the meiotic drive hypothesis. However, the possibility remains that the genotype-associated difference in number of live embryos may be due to differential fertilization or implantation. Additional breeding experiments and genotyping of pre-implantation embryos will resolve the remaining questions concerning the mechanisms involved in TRD at *R2d2*.

It is interesting to speculate about the types of embryonic lethality that are consistent with our data and with previous reports of TRD on Chr 2. Lethality is associated with distortion at *R2d2*, and thus the simplest explanation is preferential death of embryos inheriting maternal *R2d2* ^*NotWSB*^ alleles. However, such a scenario would require parent-of-origin-dependent death of embryos with maternal C57BL/6J, 129S1/SvImJ, NOD/ShiLtJ and NZO/HILtJ *R2d2* alleles in crosses involving F1 females (Table 1) and CAST/EiJ, PWK/PhJ and A/J *R2d2* alleles in the CC/DO females (S10 Fig.). The lack of evidence of TRD and parent-of-origin lethality in dozens of crosses involving these alleles [53–55], combined with the lack of evidence for imprinted genes in the central region of Chr 2 [2–4,46,56], appears to rule out this explanation. Specifically, the *Cwc22* gene present in *R2d* is not imprinted in brain, kidney, lung and liver in crosses involving the WSB/EiJ, PWK/PhJ and CAST/EiJ strains [46]. A more likely explanation for the joint and correlated presence of meiotic drive and lethality is that the unequal segregation of chromosomes and/or chromatids that leads to TRD in euploid embryos may also lead to increased Chr 2 aneuploidy, and thus to embryonic death (all autosomal aneuploidy is embryonic-lethal in the mouse). This would also explain the slight increase in the number of resorbed embryos observed at mid-gestation (S8 Fig.; S1 Table). This hypothesis makes the testable prediction that Chr 2 should be especially affected by aneuploidy in some dams with TRD.

Importantly, co-segregation of a deletion allele of *R2d2* and increased litter size in the DO-G13–44 pedigree demonstrates that lethality is mediated by an element within the *R2d* repeat.

### Maternal TRD at *R2d* is an oligogenic trait

Overall, we assessed TR at *R2d2* in hundreds of females carrying a single WSB/EiJ allele in at least nine distinct genetic backgrounds (Table 1; S1 Table). The presence of significantly different TR levels among F1 hybrid dams, combined with the fact that we observe both extreme TRD and no distortion in the progeny of females with A/J, C57BL/6J, 129S1/SvImJ, NOD/ShiLtJ, CAST/EiJ and PWK/PhJ alleles in *trans* at *R2d2* (S10 Fig.), demonstrates that TRD is under genetic control of at least one additional locus (*i*.*e*., there is at least one unlinked distorter locus that is genetically variable in the CC and DO mice). Furthermore, the presence of at least two significantly different levels of distortion among F1 hybrid dams (Table 1; S3 Fig.) indicates either that more than one distorter locus is involved or that an allelic series exists at a single distorter locus.

Further evidence that TRD is under control of one or more unlinked distorters was provided by 15 female DO-G13–44 G1 offspring that inherited the high-copy allele. Those dams had significantly different levels of TRD (*p* = 9.8x10^–5^). Note that there was no correlation between the presence or level of TRD and the paternally inherited allele (one-way ANOVA, *F* = 2.21 on 1 and 23 df, *p* = 0.15; Fig. 3).

In the DO-G13–44 pedigree, females that inherited the *R2d2* ^*WSBdel1*^ allele had copy number 11 (S11 Fig.), indicating a partial rather than complete deletion of the expansion. Using the TaqMan assay, we identified two additional DO females (DO-G13–49 and DO-G16–107; S4 Fig.; S11 Fig.) that had results consistent with a copy number loss in the WSB/EiJ haplotype. The presence of the deletion in the respective germlines was confirmed by the TaqMan assay in their progenies (S4 Fig.). Importantly, each one of the three deletions appears to be independent because these females are not closely related, their WSB/EiJ haplotypes in Chr 2 are different and the copy number present in each female is also different (S12 Fig.). The deletions appear to be internal to *R2d2* based on the analysis of the MegaMUGA genotypes and intensities [57] at all surrounding markers. The repeated observation of independent deletions indicates that *R2d2* is rather unstable and may explain the fact that, despite its presence in laboratory strains and wild mice, it has not led (yet) to a complete selective sweep.

Known meiotic drive systems (S1 Fig.) consist of one or more responder loci (a locus subject to preferential segregation during meiosis) and a single distorter (the effector locus required for drive at the responder*)*. In meiotic drive systems that are stable in natural populations, responder and distorter loci are tightly linked and are typically protected from decoupling by factors that inhibit recombination, such as structural variation [7,11,14]. Although *R2d2* resides within a recombination-cold region, the distorter is not closely linked to *R2d2* based on the TR observed and the diplotypes present in F1 hybrid and DO dams (Fig. 1; S8 Fig.). Therefore, at least one unlinked distorter is required to explain the observed variability in TRD.

These observations indicate that the maternal TRD phenotype has a complex genetic architecture. Specifically, a minimum number of copies of *R2d* are required in heterozygosity at *R2d2* for TRD to be observed. Therefore, it can be classified as overdominant, restricted to the female germline and caused by structural variation. Similar characteristics have been recently reported for the *Xce* locus that controls X-inactivation choice; notably, characterization of *Xce* relied on the analysis of a genetically diverse set of F1 hybrid mice [58]. In addition, multiple alleles at unlinked loci interact to determine whether distortion occurs at *R2d2*, and to what extent. This is unique among meiotic drive systems (S1 Fig.) and has important implications for the natural history of the system and for the ease of genetic dissection. We hypothesize that variation in TR levels at *R2d2* results from the interaction of alleles originating from multiple taxa, and thus the use of inter-specifc and inter-subspecific mouse populations was key to the characterization of this system. Wild-derived strains and wild-caught mice have enabled important biological discoveries [4,59], and we echo previous encouragements of a more prominent role for these resources in biological and biomedical research [60,61].

### What is the mechanism by which *R2d2* ^*WSB*^ influences its own segregation?

Centromeres (*i*.*e*., the site of kinetochore formation) are remarkable loci that control, in *cis*, proper segregation of chromosomes during mitosis and meiosis. It is easy to envision how a responder at, or tightly linked to, a centromere can influence chromosome segregation. Recent evidence shows that kinetochore protein levels and microtubule binding are positively correlated with preferential segregation to the oocyte in mice that are heterozygous for Robertsonian fusions [62], indicating that differences in centromere “strength” lead to meiotic drive. Responders located far away from centromeres are thought to influence their own segregation in *cis* by becoming “neocentromeres” and taking advantage of the inherited functional polarity of the female meiotic spindle [63]. We hypothesize that *R2d2* may act as a neocentromere after epigenetic activation mediated by C57BL/6J, NZO/ShiLtJ, 129S1/SvImJ, and NOD/HILtJ alleles at the distorter(s).

The discovery of multiple *R2d2* alleles with different copy numbers demonstrates that the presence of the distal insertion of *R2d* is not sufficient for meiotic drive; rather, some minimum copy number (> 11) is required for TRD. This raises the possibility that meiotic drive at *R2d2* is dosage-dependent, such that fine-scale control over the level of TRD is possible by adjusting the number of copies of *R2d*. If *R2d2* is acting as a neocentromere, this may also indicate that some minimum size and/or number of repeats is required for recognition and activation by the epigenetic machinery. The Ab10 system of maize provides examples of responders that function as neocentromeres and for which the level of meiotic drive depends on the size of the responder (*i*.*e*., knob size) [11].

The effect on the Chr 2 centromere of activating an ectopic neocentromere at *R2d2* is unknown, but it might explain the moderate levels of lethality caused by aneuploidy and suggests that some coordination between the two loci is required to achieve chromosome segregation. Meiosis involving chromosomes with neocentromeres may lead to an increased rate of non-disjunction and a reduced rate of recombination.

### Implications of *R2d2* for the CC and DO

The conclusion that a genetically complex meiotic drive system is responsible for TRD favoring the WSB/EiJ allele at *R2d2* is fully consistent with the initial observations of TRD in the CC, with our prediction that positive selection of the WSB/EiJ allele occurred during outcrossing or in early inbreeding generations [35], with the presence of similar levels of TRD in extinct and extant CC lines at intermediate generations of the CC (S5 Table) and with the fact that C57BL/6J, 129S1/SvImJ, NOD/ShiLtJ and NZO/HILtJ haplotypes at *R2d2* are not underrepresented among the currently completed CC strains (http://csbio.unc.edu/CCstatus/index.py). The observed levels of TRD in crosses that use DO females are consistent with presence of different alleles at the distorter(s) (S7 Fig.; S1 Table).

Although the discovery and identification of TRD that emerged from the DO pseudo-randomized mating scheme offered the opportunity to characterize a novel meiotic drive responder, the existence of such a locus could negatively impact the utility of this population for genetic studies. Fortunately, the locus was discovered before complete fixation of the *R2d2* ^*WSB*^ allele. Although the candidate interval spans 900 kb, TRD affects a much larger region in the DO because the strength of selection in favor of the WSB/EiJ allele is outpacing the rate at which recombination can degrade linkage disequilibrium in the region. Ultimately, this region would become an actual or statistical ‘blind-spot’ in the DO, such that the non-WSB/EiJ allele frequencies would become too small to detect allelic effects on phenotypic variation. Efforts are underway to purge the WSB/EiJ allele from the DO breeding population at this locus or to select for mice carrying a WSB/EiJ haplotype with a low copy number for *R2d2*, rather than allow the region to become fixed. Using marker-assisted selection, progeny of heterozygous WSB/EiJ carrier crosses are excluded from subsequent generations. Allele frequencies and random segregation on all other chromosomes are being preserved (EJC unpublished).

### Concluding remarks

The SPRET/EiJ and WSB/EiJ strains and the Hsd:ICR outbred stocks are among the most extensively characterized and utilized mouse populations. Resources involving those populations include whole-genome sequencing and genotyping [24,37], development of linkage maps of the mouse [40,64,65], creation of genetic reference populations [35,36,66], experimental crosses to map a diverse collection of biomedical and evolutionary traits [33,48,53,61] and selection lines derived from Hsd:ICR (such as M16i and HR) that have been widely used for genetic analyses [30–32,67–69]. The potential for distorted allele frequencies in crosses involving those populations may affect the interpretation of results from a wide range of genetic, behavioral and physiological studies.

The *R2d2* system has attributes that make its genetic and mechanistic characterization a tractable problem. Identification of several distorters would allow assembling the pathway(s) responsible for centromere function and spindle polarity. This may open the way to explore at the molecular and mechanistic levels an evolutionary force (meiotic drive) thought to be responsible for karyotype evolution in mammals and in many other organisms [15]. With the advent of genome engineering tools such as CRISPR/Cas9 [70], we also anticipate practical applications of a strong, modulable meiotic drive system with only modest levels of lethality. For example, meiotic drive could be used to increase the efficacy of gene drives for introducing new genes into experimental or natural populations [71].

## Materials and Methods

### Ethics statement

All animal work was performed according to one of the following protocols: 1) the *Guide for the Care and Use of Laboratory Animals* under approved IACUC animal use protocols within the AAALAC accredited program at the University of North Carolina at Chapel Hill (Animal Welfare Assurance Number: A-3410–01); 2) the requirements of The Jackson Laboratory Animal Ethics Committees under approved protocol #JAX10001; 3) an animal protocol approved by the North Carolina State University Institutional Animal Care and Use Committee (09–0133-B); or 4) an animal study protocol approved by the NCI Animal Care and Use Committee (ASP# LCBG-013). All animals were euthanized according to the regulations of the governing protocol.

### Published mouse crosses

The G2:F1 population has been previously reported and was genotyped on the Mouse Diversity Array [72] (MDA). A population of 96 (FVB/NJx(WSB/EiJxPWK/PhJ)F1)G2 mice was previously reported and was genotyped on the MegaMUGA array [40,53]. DNAs from selected progeny from previously published (C57BL/6JxSPRET/EiJ)xC57BL/6J and (A/JxSPRET/EiJ)xA/J backcrosses [26,43] were regenotyped on the MegaMUGA array. The SPRET/EiJ strain designation had not yet been assigned to the inbred strain at the time the backcross was performed [26]. Finally, DNA from multiple samples from the (M16ixL6)F2 intercrosses and from generations 4 and 10 of the (HR8xC57BL/6J) advanced intercross line [29,31,32] were genotyped at markers closely linked to *R2d2*.

### New mouse crosses

**Crosses 1–2, 7–10 and 16–17 (Table 1)**. WSB/EiJ and C57BL/6J were used in reciprocal combinations. Male F1 hybrids were backcrossed to C57BL/6J to produce the progeny of crosses 1 and 2. Female F1 hybrids were backcrossed to C57BL/6J to produce the progeny of crosses 16 and 17. The progeny of crosses 7–10 was produced in a similar way to crosses 16 and 17, except that female F1 of reciprocal matings of WSB/EiJ and CAST/EiJ were used for crosses 7 and 8, and female F1 of reciprocal matings of WSB/EiJ and PWD/PhJ were used for crosses 9 and 10. All breeding was done at the Jackson Laboratory (Bar Harbor, ME).

**All other crosses**. DO mice and standard mouse inbred strains (129S1/SvImJ, A/J, C57BL/6J, CAST/EiJ, FVB/NJ, NU/J, NOD/ShiLtJ, NZO/H1LtJ, PWK/PhJ and WSB/EiJ) were obtained from The Jackson Laboratory (Bar Harbor, ME). CC mice were obtained from the Systems Genetics Core Facility colony at UNC Chapel Hill [73] (http://csbio.unc.edu/CCstatus/index.py). Those mice were used to generate the following number and types of hybrid mice: nine (129S1/SvImJxWSB/EiJ)F1 females; two (A/JxWSB/EiJ)F1 females; seven (NOD/ShiLtJxWSB/EiJ)F1 females; six (NZO/HILtJxWSB/EiJ)F1 females; 10 (CC042/GeniUncxCC001/Unc)F1 females; three (CC001/UncxCC039/Unc)F1; nine (DOxCC001/Unc)F1 females, 13 (DOxCC005/Tau Unc)F1 females and five (NU/JxC57BL/6J). F1 females were mated to FVB/NJ males and cages were surveyed three to five times per week. Litter sizes were recorded and pups were sacrificed at birth, and tissue was collected for DNA isolation. The same breeding schema was followed with 127 DO *R2d* heterozygous females used to determine the origin of maternal TRD. All breeding was done at UNC Chapel Hill (Chapel Hill, NC).

### Linkage mapping of TRD in DO-G13–44xCC cross

A single G13 DO female (DO-G13–44) was mated to a male that was the result of an intercross between four CC lines (CC013/GeniUnc, CC053/Unc, CC065/Unc and CC008Geni/Unc; Fig. 4). G3 female progeny were weaned, single housed and mated to FVB/NJ males. Cages were surveyed three to five times per week. Litter sizes were recorded and G4 pups were sacrificed at birth, and tissue was collected for DNA isolation.

TR was measured in G3 dams as described above. Each dam was classified as having TRD (*p* < 0.05 for 1-df *Χ* ^*2*^ test of null hypothesis TR = 0.5) or not having TRD (*p* ≥ 0.05). Both G2 parents and G3 dams were genotyped on MegaMUGA and phased haplotypes at *R2d2* were inferred by manual inspection of haplotype reconstructions. In order to isolate the contribution of maternal and paternal alleles to TRD, MegaMUGA markers called as H in the G2 dam and homozygous in the G2 sire were retained for mapping, and presence of TRD was mapped as a binary phenotype using a logistic regression analog to the Haley-Knott method. The procedure was repeated using only markers called as H in the father of the G3 dams and homozygous in the mother. Significance thresholds for LOD scores were obtained by unrestricted permutation.

### DNA isolation and genotyping

**Crosses 1–2, 7–10 and 16–17 (Table 1)**. DNA was prepared from spleens of 21-day old mice. DNA extraction and SNP genotyping were carried out as described previously [74].

**All other samples**. DNA for PCR-based genotyping was performed on crude whole genomic DNA extracted by heating tissue in 100ul of 25mM NaOH/0.2mM EDTA at 95°C for 60 minutes followed by the addition of 100ul of 40mM Tris-HCl. The samples were then spun at 2000 rpm for 10 minutes and the supernatant collected for use as PCR template. All primers (S6 Table) used in this study were designed using PrimerQuest software (https://www.idtdna.com/Primerquest). PCR reactions contained 1.5–2 mM MgCl2, 0.2–0.25 mM dNTPs, 0.2–1.8 μM of each primer and 0.5–1 units of GoTaq polymerase (Promega) in a final volume of 10–50 μL. Cycling conditions were 95°C, 2 min, 35 cycles at 95°, 55° and 72°C for 30 sec each, with a final extension at 72°C, 7 min. PCR products were loaded into a 2% agarose gel and run at 200 V for 40–120 minutes (depending on the marker). Genotypes were scored and recorded.

DNA for MegaMUGA genotyping was isolated as described previously [40,53]. Briefly, ~2 mm of mouse tail (5 mg) was harvested, flash-frozen on dry ice and digested with proteinase K overnight at 65°C. The following day, DNA was extracted using the QIAGEN Puregene Gentra kit (kit no. 158389; QIAGEN GmbH, Hilden Germany). Genotyping was performed with the MegaMUGA genotyping microarray (Neogen/GeneSeek, Lincoln, NE), a 78,000-probe array based on the Illumina Infinium platform.

**Genotyping by TaqMan**. After *R2d2* was established as the causal variant for TRD, a subset of DO-G16 progeny and all (M16i x L6)F2 intercross progeny were genotyped using TaqMan real-time PCR assays for *Cwc22*. Samples heterozygous for a high-copy allele at *R2d2* can be readily distinguished from samples homozygous for a low-copy allele based on the normalized cycle threshold value estimated from the assay (see section “Copy-number validation” below).

### Statistics

**Deviation from Mendelian transmission**. TR is reported as the ratio of the WSB/EiJ genotype to the total number of genotypes: WSB / (WSB + nonWSB). *P* values for aggregate data were calculated using a *Χ* ^*2*^ goodness-of-fit test of the observed number of WSB/EiJ genotypes compared to the number of WSB/EiJ genotypes expected under the null hypothesis of equal transmission:

$$X^{2}=\frac{{\left(WSB-\frac{WSB+nonWSB}{2}\right)}^{2}}{\frac{WSB+nonWSB}{2}}$$

For individual dams, the small sample sizes (typically fewer than 50 total offspring) would lead to type II error; therefore, *p*-values were calculated using an exact binomial test. Confidence intervals for TRs were calculated using the binom R package (http://cran.r-project.org/web/packages/binom/).

**Average litter size**. Average litter size was calculated as the mean number of offspring counted soon after birth per litter per dam (± standard deviation), including the number of viable embryos counted *in utero* in mid-gestation DO dams (unless otherwise noted).

The expected average litter size (ALS) of a dam under a model in which lethality is the sole explanation for TRD is:

$$ALS_{Obs}=ALS_{Exp}(1-\frac{2TR-1}{2TR})$$,

where *ALS*_*Exp*_ is the mean ALS in dams with no TRD [41]. Significance of the deviation of *ALS*_*Obs*_ from *ALS*_*Exp*_ was determined using a Wilcox signed rank test.

**Inheritance of *R2d2* alleles**. Similarly, the average absolute number of offspring inheriting each *R2d2* allele was calculated as the mean number of offspring per litter per dam having each of the possible genotypes. Significance was determined using a one-tailed Student *t*-test.

### Estimation of embryonic lethality

DO and F1 dams were euthanized by CO_2_ asphyxiation 12–18 days after delivery of the previous litter and the uterus was dissected. The number of live embryos and reabsorbed (dead) embryos was recorded. Each live embryo was dissected to isolate DNA for genotyping. Tissue from each live embryo was harvested for DNA extraction and genotyping.

### Analysis of genotyping arrays

All MDA arrays were genotyped using MouseDivGeno [57], and all MegaMUGA arrays were genotyped using Illumina BeadStudio. We plotted number of H and N calls (as a fraction of the total number of genotypes) for each group of similar samples and excluded outliers from further analysis. For CC lines, DO animals, CCxCC F1 females and DOxCC F1 females, we inferred haplotypes using probabilistic methods [40,75]. As an additional QC step, we grouped DO samples by generation and plotted the number of recombinations (counted as unique transitions in haplotype reconstructions) and removed outliers.

### Linkage mapping of *R2d2*

**CAST/EiJ allele in the CC G2:F1**. Thirty-four MDA SNP probe sets were identified within *R2d* in the GRCm38 reference sequence (S3 Table). We ensured that these probes were unique using BLAT [76] to map them to the reference genome. In order to map the expansion allele present in the CAST/EiJ strain, phenotypes and genotypes were coded as follows. First, we applied a CCS transform [77] to the mean intensity of all probes in each probe set using MouseDivGeno [57] and summed the values for each sample to obtain the final phenotype value. Next, the genome was divided into a set of disjoint intervals whose boundaries were defined by the 21,933 unique recombination events inferred in the population [40], so that no individual would be recombinant within any of the resulting intervals. Then, using haplotype reconstructions, individuals were coded as either heterozygous (CAST/not-CAST) or homozygous (not-CAST/not-CAST) within each interval (there are no CAST homozygous individuals in this population). Of 474 individuals, 144 with a WSB/EiJ allele in the middle of chromosome 2 were excluded to yield a final sample size of 330. A single-locus QTL scan was then performed via Haley-Knott regression [78], treating the population as a backcross.

**WSB/EiJ allele in an intercross population**. Three MegaMUGA SNP probes were identified within *R2d* in the GRCm38 reference (S4 Table). Again, uniqueness was verified using BLAT. In order to map the expansion allele in WSB/EiJ, the sum intensity of these probes was used as a phenotype and genotypes were coded as follows. First the genome was divided into a grid of 1,000 disjoint intervals of approximately equal size, and one MegaMUGA SNP marker segregating between WSB/EiJ and PWK/PhJ was selected per interval. Individuals were coded as heterozygous (WSB/not-WSB) or homozygous (not-WSB/not-WSB) at each marker. A single-locus QTL scan was then performed using Haley-Knott regression as implemented in R/qtl [79], treating the population as a backcross.

### Fine-mapping of *R2d2*

In order to refine the location of *R2d2*, we identified individual mice with recombinant chromosomes within the candidate interval defined by linkage mapping. These *critical recombinants* define the proximal and distal boundaries of the refined candidate interval.

**CAST/EiJ allele**. We partitioned the 330 G2:F1 individuals without a WSB/EiJ allele in the *R2d* locus into two groups according to MDA sum-intensity values. From those with sum-intensity consistent with a non-CAST/EiJ expansion allele, we selected the most distal recombinants from CAST/EiJ to another haplotype. From those with sum-intensity consistent with the CAST/EiJ expansion allele, we selected the most distal recombinant from another haplotype to CAST/EiJ. Together these recombinants define the proximal boundary of the candidate interval in CAST/EiJ. Similarly, in order to define the distal boundary of the candidate interval, we selected the most proximal recombinants from CAST/EiJ to another haplotype that still had sum-intensity consistent with the CAST/EiJ expansion allele.

**WSB/EiJ allele**. The boundaries of the WSB/EiJ candidate interval were mapped in the same fashion using 229 individuals spanning generations 10 through 14 of the DO, all of which have been genotyped on MegaMUGA and are recombinant for WSB/EiJ in the initial candidate interval. We first excluded individuals homozygous for WSB/EiJ over any interval with in the interval. Then we selected the most distal recombinants from another haplotype to WSB/EiJ, which also had MegaMUGA sum-intensity values consistent with a non-WSB/EiJ expansion allele. These recombinants define the distal boundary of the candidate interval. We mapped the proximal boundary similarly.

**SPRET/EiJ allele**. (C57BL/6JxSPRET/EiJ)xC57BL/6J (*n* = 12) and (A/JxSPRET/EiJ)xA/J progeny (*n* = 17) [26,43] genotyped on the MegaMUGA array were used to refine the candidate interval for the expansion allele in SPRET/EiJ. Haplotypes in the relevant region of Chr 2 were inferred by manual inspection of genotype calls. Samples were partitioned according to sum-intensity at the three MegaMUGA SNP probes tracking the expansion allele. Among individuals with sum-intensity consistent with the expansion allele, the most proximal recombinant from SPRET/EiJ to another haplotype defines the distal boundary of the candidate interval. Likewise the most distal recombinant from a non-SPRET/EiJ haplotype to SPRET/EiJ defines the proximal boundary of the candidate interval.

### Whole-genome sequencing

Ten individuals from the HR8 selection line were selected for whole-genome sequencing. Five micrograms of high-molecular-weight DNA were used to construct TruSeq Illumina libraries, using 0.5 μg starting material, with 300- to 400- and 400- to 500-bp fragment sizes. Each library was sequenced on one lane of an Illumina HiSeq2000 flowcell, as paired-end reads, with 100-bp read lengths. We aligned the sequences to the University of California at Santa Cruz Mouse Build mm9. HR8 sequenced reads were aligned to the mouse genome (mm9) using bowtie 2.2.3 [80] with default options. We removed PCR duplicates and filtered low-quality SNPs using samtools 0.1.19 [81] and Picard 1.88 (http://picard.sourceforge.net/).

### Sequence variants and read depth

We retrieved BAM files of aligned reads (Oct 2012 release) from the Sanger Mouse Genomes Project FTP site (ftp-mouse.sanger.ac.uk). We used the mpileup function of samtools [81] to call sequence variants on the HR8 and Sanger BAM files jointly and to output the read depth at each base. We counted a SNP as private to WSB/EiJ, SPRET/EiJ and the 10 HR8 individuals if those samples all shared a genotype that was different from the seven other CC founder strains. We defined the boundaries of the copy number expansion by identifying consecutive 100bp windows in which the average read depth was at least twice the genome-wide average read depth. We estimated the number of copies of the expansion as the modal per-base read depth.

### Copy number validation

We used commercially-available TaqMan assays for *Cwc22* to estimate the copy number of *R2d2*. We used two copy number assays (Life Technologies catalog numbers Mm00644079_cn, Mm00053048_cn) to target the number of *Cwc22* copies (proximal and distal). We also used two reference assays (*Tfrc*, cat. no. 4458366, for target Mm00053048_cn; *Tert*, cat. no. 4458368, for target Mm00644079_cn), for genes known to exist in a single haploid copy in the mouse, to calibrate the amplification curve. Assays were performed according to the manufacturer’s protocol on an ABI StepOne Plus Real-Time PCR System (Life Technologies, Carlsbad, CA). Cycle thresholds (Ct) for each assay were determined using the ABI CopyCaller v2.0 software with default settings. For each target-reference pair, relative cycle threshold (ΔCt) was calculated as

$$\Delta C_{t}=C_{{}_{t}}^{reference}-C_{t}^{target}$$

The ΔCt value is proportional to copy-number of the target gene on the log scale but is subject to batch effects. In order to account such effects, normalized ΔCt values for each sample were calculated as follows. A standard set of control samples (from C57BL/6J, WSB/EiJ, CAST/EiJ and (WSB/EiJxC57BL/6J)F1 mice), spanning the expected copy-number range for *Cwc22*, were included in duplicate or triplicate in every assay batch. A linear mixed model was fit to raw ΔCt values for these control samples, with target-reference pair and batch as random effects, using the lme4 package (http://lme4.r-forge.r-project.org/) for R (http://www.R-project.org/). Predicted values (best linear unbiased predictors, BLUPs) from this model capture technical variation orthogonal to variation due to genotype. BLUPs calculated from control samples were subtracted from raw ΔCt values for all samples, and the residual was used as the normalized ΔCt for copy-number estimation.

In this manuscript we chose in most cases to present ΔCt, rather than extrapolated absolute copy number, because ΔCt is the natural scale of the data (*i*.*e*., the log scale). Constant variance (with respect to mean) on the log scale grows exponentially on the linear scale so that estimates of absolute copy number become increasingly uncertain as copy-number grows.

### Linkage mapping of *Cwc22* TaqMan assay

The use of TaqMan assays for *Cwc22* as a proxy for copy number at *R2d2* was validated by mapping normalized ΔCt for target Mm00644079_cn as a quantitative phenotype in 64 members of the (FVB/NJx(WSB/EiJxPWK/PhJ)F1)G2 intercross population described above. The marker selection and mapping procedure were the same as described above for mapping MegaMUGA sum-intensity values.

### Availability of data

Chr 2 genotypes and whole-genome sequence that have not been published elsewhere are available at http://csbio.unc.edu/r2d2.

## Supporting Information

S1 Fig Overview of known meiotic drive systems. Data for 13 meiotic drive systems are shown. Each box represents the maximum TR observed in each system. Dotted lines indicate variability in observed TRs. For each system, the lower panel provides the type of *responder* locus; the species in which it was identified; the range of observed TRs; whether *distorter* loci are known, and if so how many and whether they are linked to the *responder*. Meiotic drive of *Om* in DDK and an HSR in wild *M*. *m*. *musculus* mice is dependent on the genetic background of the sperm that fertilize the egg. All images are from wikipedia.org (Creative Commons license).
(TIF)

S2 Fig The WSB/EiJ allele is significantly overrepresented in the Diversity Outbred (DO) population. Allele frequencies of the eight CC founder alleles in 1,175 individuals from generation eight of the DO are shown at 1 Mb intervals on Chr 2. The expected frequency of 0.125 is shown as a dashed line. The boundaries of the *R2d* candidate interval are shown by the yellow box, and the boundaries 900 kb interval where copy number expansion has occurred is shown by the blue box.
(TIF)

S3 Fig Three distinct classes of transmission ratio (TR) in progeny of F1 hybrid parents. TRs are shown for all crosses in Table 1 (red circles). Boxplots show the ranges of TRs observed in four sets of crosses (numbered according to Table 1): heterozygous sires (1–6) and heterozygous dams with no TRD (7–10), intermediate TRD (11–15) and high TRD (16–18). The first two classes are not different from the Mendelian expectation of 0.5, nor from each other. The third and fourth classes are significantly different from 0.5, from each other, and from the first two classes.
(TIF)

S4 Fig Linkage mapping localizes *R2d2* to a 900 kb region in Chr 2. The recombinant haplotypes and sum intensities A) for 34 MDA probes in 58 mice defining the boundaries of copy-number gain in the CAST/EiJ strain, and B) 3 MegaMUGA probes in 74 mice defining the boundaries of copy-number gain in the WSB/EiJ strain. Haplotypes are colored as in the legend in S2 Fig.. C) Distribution of sum-intensity for the three probes in the *R2d2* copy number gain region present on MegaMUGA for offspring of a (C57BL/6JxSPRET/EiJ)F1xC57BL/6J (BSB) backcross or a (A/JxSPRET/EiJ)F1xA/J (ASA) backcross is shown in the top right panel, and the sum intensities and recombinant haplotypes in the mice are shown below. Haplotypes are colored by parental strain: SPRET/EiJ (brown), C57BL/6J (black) or A/J (yellow). The high sum intensity, associated with a copy number gain, that is present in ASA-74-B localizes *R2d2* distal to the location of the unique copy in the reference sequence (gray dotted line).
(PDF)

S5 Fig *R2d2* copy number in dams tested for TR and in their progenies. Normalized ΔC_t_, normalized cycle threshold by TaqMan qPCR assay (see Methods). A) Homozygous calibration samples used for TaqMan assays targeting *Cwc22*: C57BL/6J (dark grey), haploid copy number 1; CAST/EiJ (green), copy number 2; (WSB/EiJxC57BL/6J)F1 (lavender), copy number ~17; and WSB/EiJ (purple), copy number ~33. In panels B-H, all samples are predicted to be heterozygous for the *R2d2* ^WSB^ allele based on genotype by PCR at marker Chr2:85.65Mbp. B) F1 hybrids between inbred CC lines used to define the 9.3 Mb candidate interval (see S1 Table). C) G1 hybrids between DO females and CC males used to define *R2d* candidate interval (see S1 Table). D) Heterozygous DO G13 dams. Outlier sample marked in red is female DO-G13–049, dam of samples in panel G. Sample marked in red and with (*) is female DO-G13–044, the dam of samples in panel H. E) Progeny of DO G13 dams according to predicted copy number (CN), based on TaqMan assay of corresponding G13 dam. Red points are progeny of female DO-G13–049. F) G3 progeny of family DO-G13–44 (see Fig. 3), the offspring of female DO-G13–044, according to predicted CN based on haplotypes linked to *R2d*. G) G4 progeny in family DO-G13–44, according to predicted CN based on TaqMan assay of corresponding G3 dams. Only low-molecular weight (LMW) DNA was available for samples in panels G and I; note that ΔC_t_ values obtained from LMW DNA are not directly comparable to ΔC_t_ values from high-molecular weight DNA. H) Heterozygous DO-G16 dams. Outlier sample marked in red is DO-G16–107, dam of samples in panel I. I) Progeny of DO-G16 dams according to predicted copy number (CN), based on TaqMan assay of corresponding DO-G16 dam. Red points are progeny of DO-G16–107.
(PDF)

S6 Fig TR is variable in DO and CC females. TRs (points) and 95% confidence intervals (lines) for each female from the different types of crosses indicated in the legend. Gray points represent crosses between heterozygous DO females and FVB/NJ males. All other crosses are those that appear in Fig. 1. Females with a mutant *R2d2* ^*WSB*^ allele are excluded. Dotted line shows Mendelian expectation of 0.5.
(PDF)

S7 Fig Lethality is not sufficient to explain observed TRD. The ratio of observed to expected litter size [(*ALS*_Exp_—*ALS*_*Obs*_) / *ALS*_*Exp*_] under a model in which TRD is explained solely by lethality (see Materials and Methods) is shown for TRs between 0.5–1.0 (black line), where expected litter size is 8.4 (the mean litter size of DO females without TRD). Dotted lines show the relationship between lethality and TR for three representative TR values (0.92 represents the threshold used in the text to define DO females with extreme TRD); for each value, the expected ratio and equivalent litter size are shown. Colored squares show aggregate values for three of the test crosses shown in Table 1: light blue = cross 12, (NOD/ShiLtJxWSB/EiJ)F1; pink = cross 13, (129S1/SvImJxWSB/EiJ)F1; dark blue = cross 15, (NZO/HlLtJxWSB/EiJ)F1. Other shapes show values for individual DO females (identified with “**” in S1 Table). Females with a mutant *R2d2* ^*WSB*^ allele are excluded. Note that females below the black line have TRs that are too high to be explained solely by lethality given their average litter sizes.
(PDF)

S8 Fig Embryonic lethality at midgestation. Count of dead embryos per dam at mid-gestation in heterozygous dams. Filled points, dams with TRD; open points, dams with no TRD. Points are jittered to reveal coincident values.
(PDF)

S9 Fig Effect of *R2d2* genotype on ovulation rates and live embryos in (M16i x L6)F2 females. Ovulation rate, in oocytes per dam A), and count of live embryos per dam B), according to genotype at *R2d2*, assayed by TaqMan. Genotypes are coded as LL = homozygous L6, ML = heterozygous, MM = homozygous M16i.
(PDF)

S10 Fig Chr 2 haplotypes in DO and CC females. Chr 2 haplotypes in *R2d2* heterozygous dams assessed for TR. Shown colored by CC founder strain (see legend in S2 Fig.) are the haplotypes found in *cis* (left panel) and *trans* (right panel) to the WSB/EiJ allele in females A) with TRD and B) without TRD. Females with a mutant *R2d2* ^*WSB*^ allele are excluded. The black box shows the boundaries of the *R2d2* candidate interval.
(PDF)

S11 Fig Chr 2 haplotypes in DO females heterozygous for a copy-number loss at *R2d2*. Haplotypes are shown colored by CC founder strain (see legend in S2 Fig.). White Δ indicates location of deletion. Phasing is arbitrary except in DO-G13–044 (G2 dam in family DO-G13–44), whose haplotypes could be phased by manual inspection of offspring genotypes. Copy number at the *R2d2* locus for each chromosome (estimated from TaqMan normalized ΔC_t_ values in progeny bearing that chromosome) is indicated at right: first the best estimate of integer copy number, then mean of point estimates across progeny ± 1 standard error.
(PDF)

S12 Fig TR and copy number at *R2d2* in the progeny of (NU/JxC57BL/6J)F1 and DO-G16 females. Filled points, heterozygous samples; open points, homozygous control samples. Progeny can be clearly divided into two classes (high normalized ΔC_t_, NU/J or WSB/EiJ allele; low normalized ΔC_t_, alternate allele), demonstrating that the TaqMan assay is appropriate for genotyping at *R2d2*. Progeny of additional DO-G13 and DO-G16 samples suspected to carry low-copy alleles are shown for comparison.
(PDF)

S1 Table Transmission ratio and litter size in *R2d2* ^*WSB/NotWSB*^ heterozygous DO, CCxDO and CCxCC dams. For each dam, the numbers of offspring having each of the two possible genotypes is shown, along with their ratio (TR) and *p*-value for a one-sided exact binomial test of deviation from the Mendelian expectation of 0.5; the average litter size (ALS) ± standard deviation (ALS.SD); the number of live/resorbed embryos counted *in utero* at mid-gestation of the final litter; the 95% confidence interval (CI) of the TR; the average number of WSB/EiJ and non-WSB/EiJ alleles per litter; the maximum litter size; the TaqMan ΔC_t_ value, standard deviation, and predicted copy number; and the TRD classification: N = no TRD (TR < 0.6 or *p* > = 0.1), L = low TRD (TR ≥ 0.6 and *p* > 0.05), M = intermediate TRD (*p* ≤ 0.05, 0.6 ≥ TR < 0.92), H = high TRD (TR ≥ 0.92); U = unclassified (sample size < 10), X = low copy number due to deletion. Females used in determining the *R2d2* candidate interval (Fig. 1) are shown in bold.
(XLSX)

S2 Table Reference sequence positions of copy number expansion in WSB/EiJ and SPRET/EiJ. Start and end positions (NCBI/37) and sizes are shown for the regions of the reference sequence exhibiting copy number gains.
(XLSX)

S3 Table MDA markers within *R2d* used for sum intensity calculations. (XLSX)

S4 Table MegaMUGA markers within *R2d* used for sum intensity calculations. (XLSX)

S5 Table *R2d2* Allele frequencies in CC populations. Extinct: CC lines that have gone extinct during the inbreeding process; PreCC: CC lines that are not fully inbred; Available: fully inbred CC lines that are available for purchase. Data sets have some degree of overlap. Expected allele frequency in the absence of TRD is 0.125.
(XLSX)

S6 Table Primers used for PCR genotyping. There are two rows for each amplicon, one for the forward strand primer and one for the reverse strand primer. The reference position of the first based of the primer sequence is given (NCBI/37).
(XLSX)
