# Supplementary figures and images for: A Multi-Megabase Copy Number Gain Causes Maternal Transmission Ratio Distortion on Mouse Chromosome 2
Source: PLoS Genet. 2015 Feb 13;11(2):e1004850. doi: 10.1371/journal.pgen.1004850 (PMC4334553; doi:10.1371/journal.pgen.1004850)

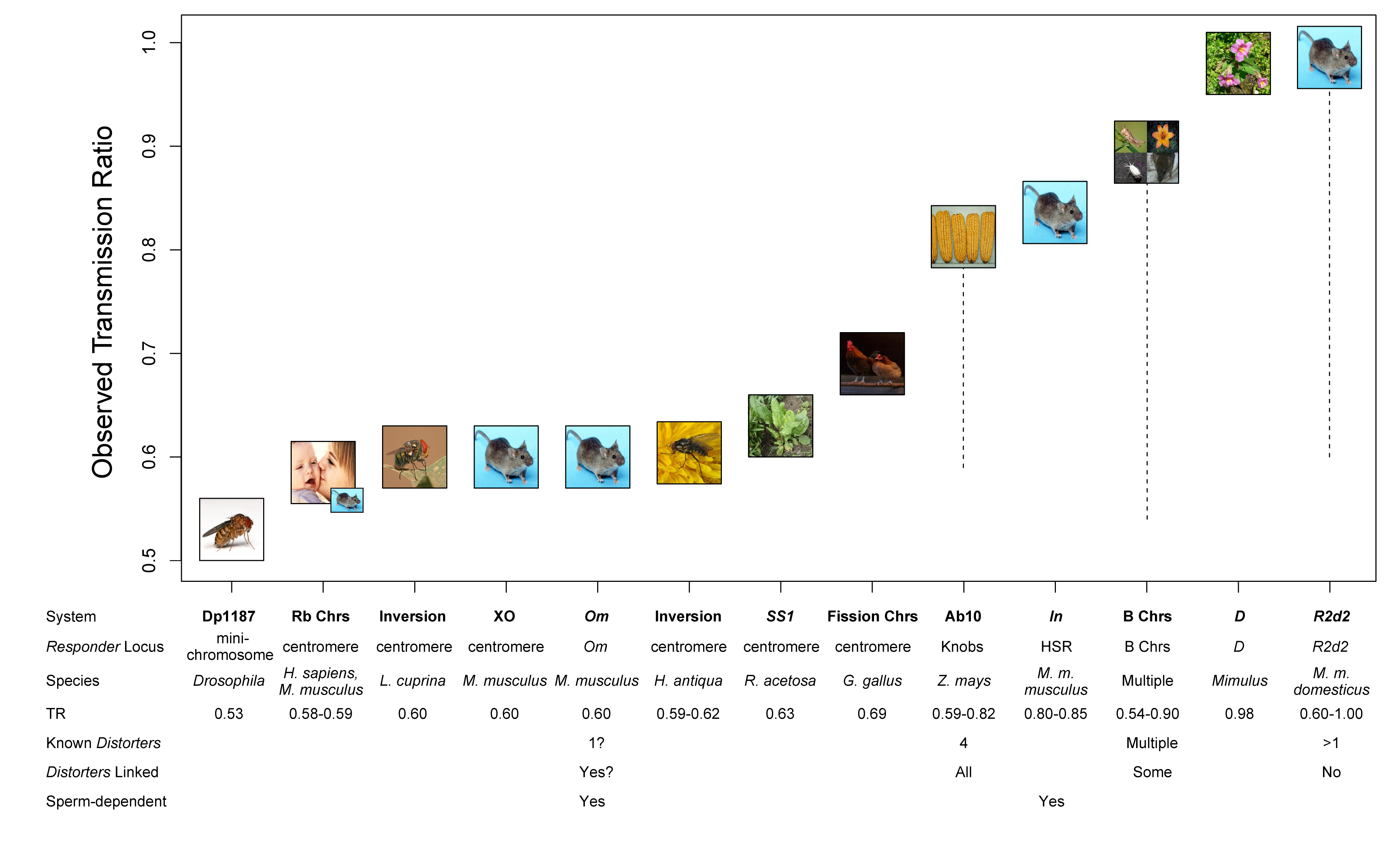

Supplement: S1 Fig — Data for 13 meiotic drive systems are shown. Each box represents the maximum TR observed in each system. Dotted lines indicate variability in observed TRs. For each system, the lower panel provides the type of responder locus; the species in which it was identified; the range of observed TRs; whether distorter loci are known, and if so how many and whether they are linked to the responder. Meiotic drive of Om in DDK and an HSR in wild M. m. musculus mice is dependent on the genetic background of the sperm that fertilize the egg. All images are from wikipedia.org (Creative Commons license). (TIF) [file pgen.1004850.s001.tif]

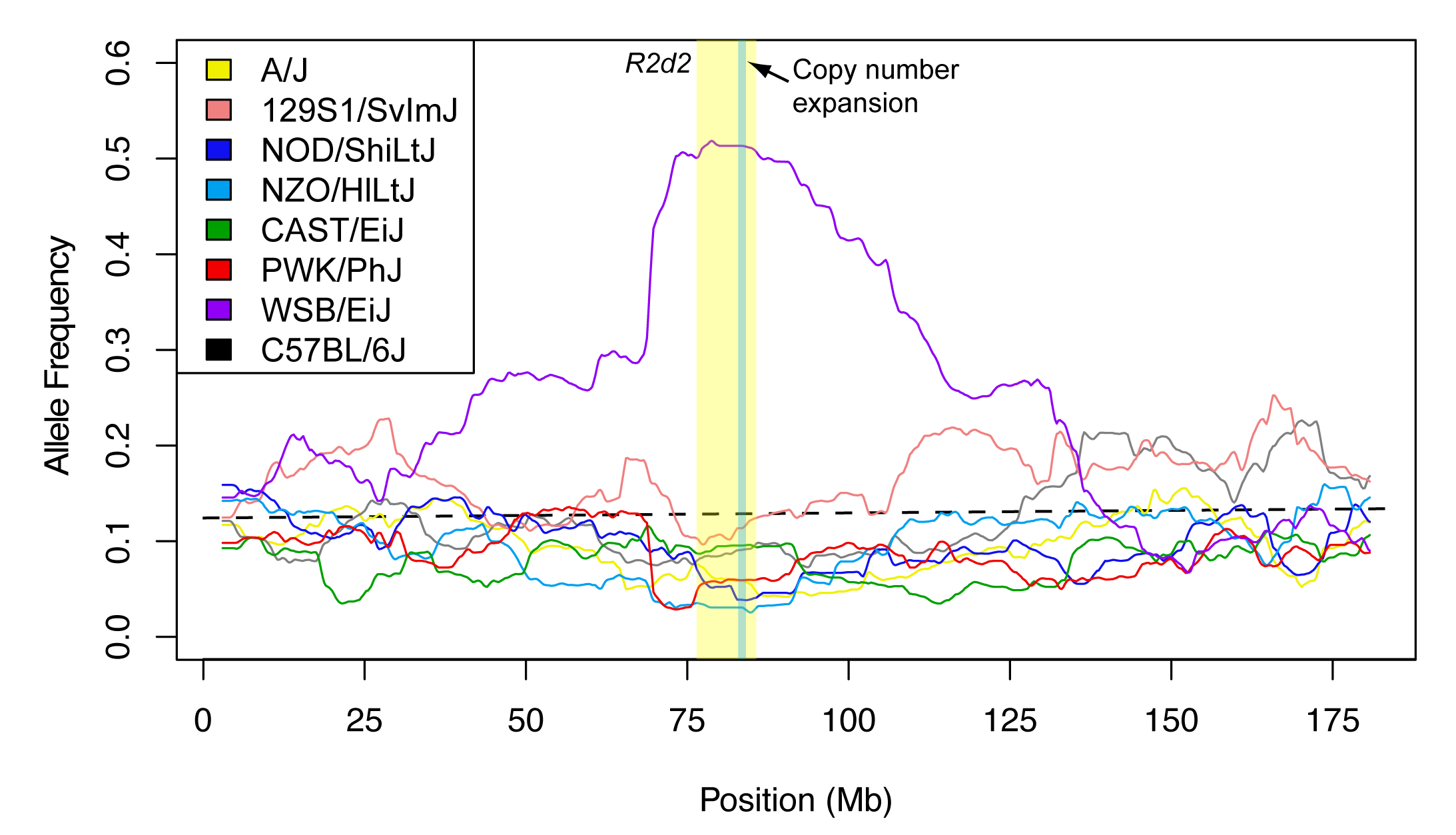

Supplement: S2 Fig — Allele frequencies of the eight CC founder alleles in 1,175 individuals from generation eight of the DO are shown at 1 Mb intervals on Chr 2. The expected frequency of 0.125 is shown as a dashed line. The boundaries of the R2d candidate interval are shown by the yellow box, and the boundaries 900 kb interval where copy number expansion has occurred is shown by the blue box. (TIF) [file pgen.1004850.s002.tif]

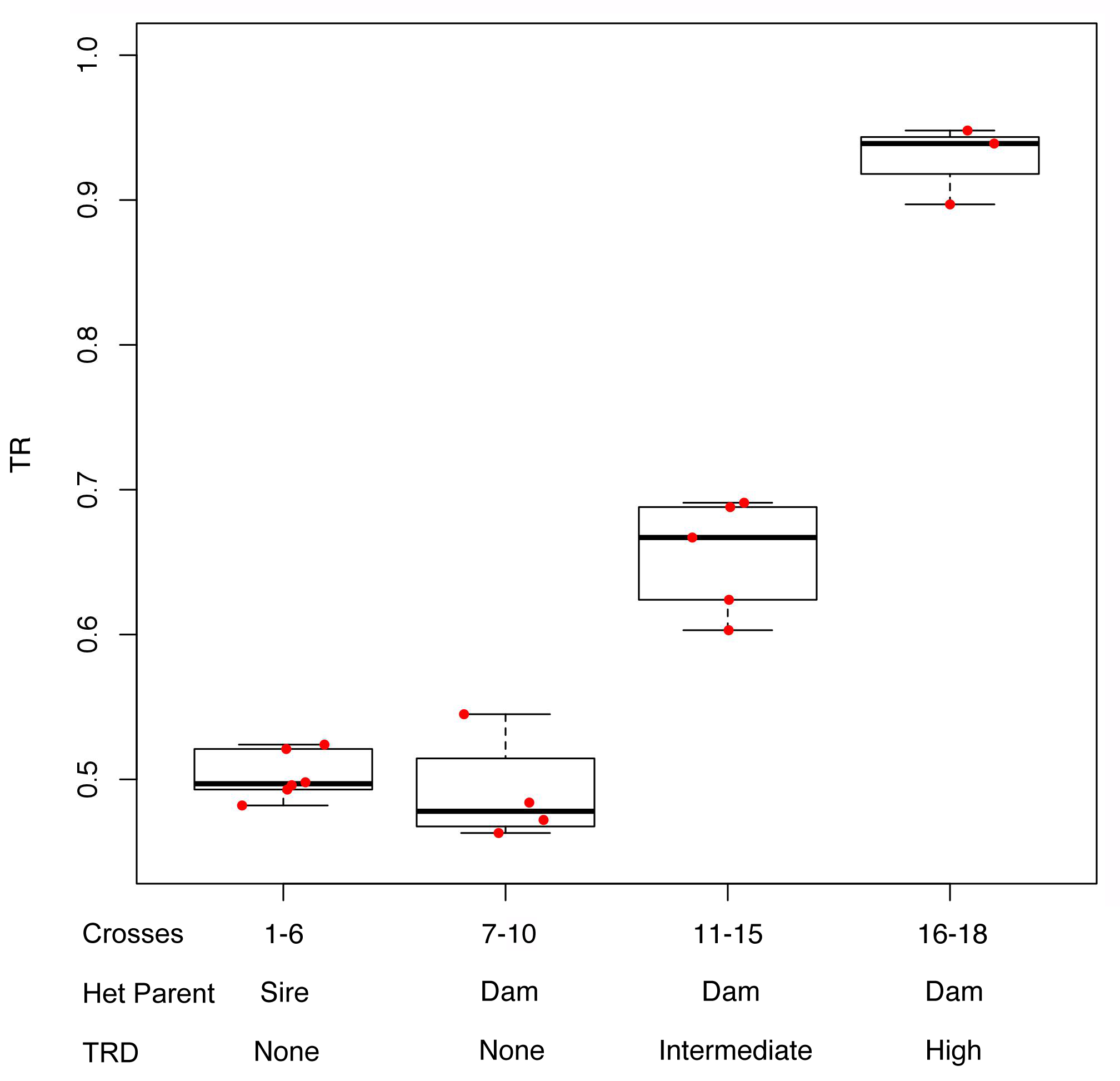

Supplement: S3 Fig — TRs are shown for all crosses in Table 1 (red circles). Boxplots show the ranges of TRs observed in four sets of crosses (numbered according to Table 1): heterozygous sires (1–6) and heterozygous dams with no TRD (7–10), intermediate TRD (11–15) and high TRD (16–18). The first two classes are not different from the Mendelian expectation of 0.5, nor from each other. The third and fourth classes are significantly different from 0.5, from each other, and from the first two classes. (TIF) [file pgen.1004850.s003.tif]

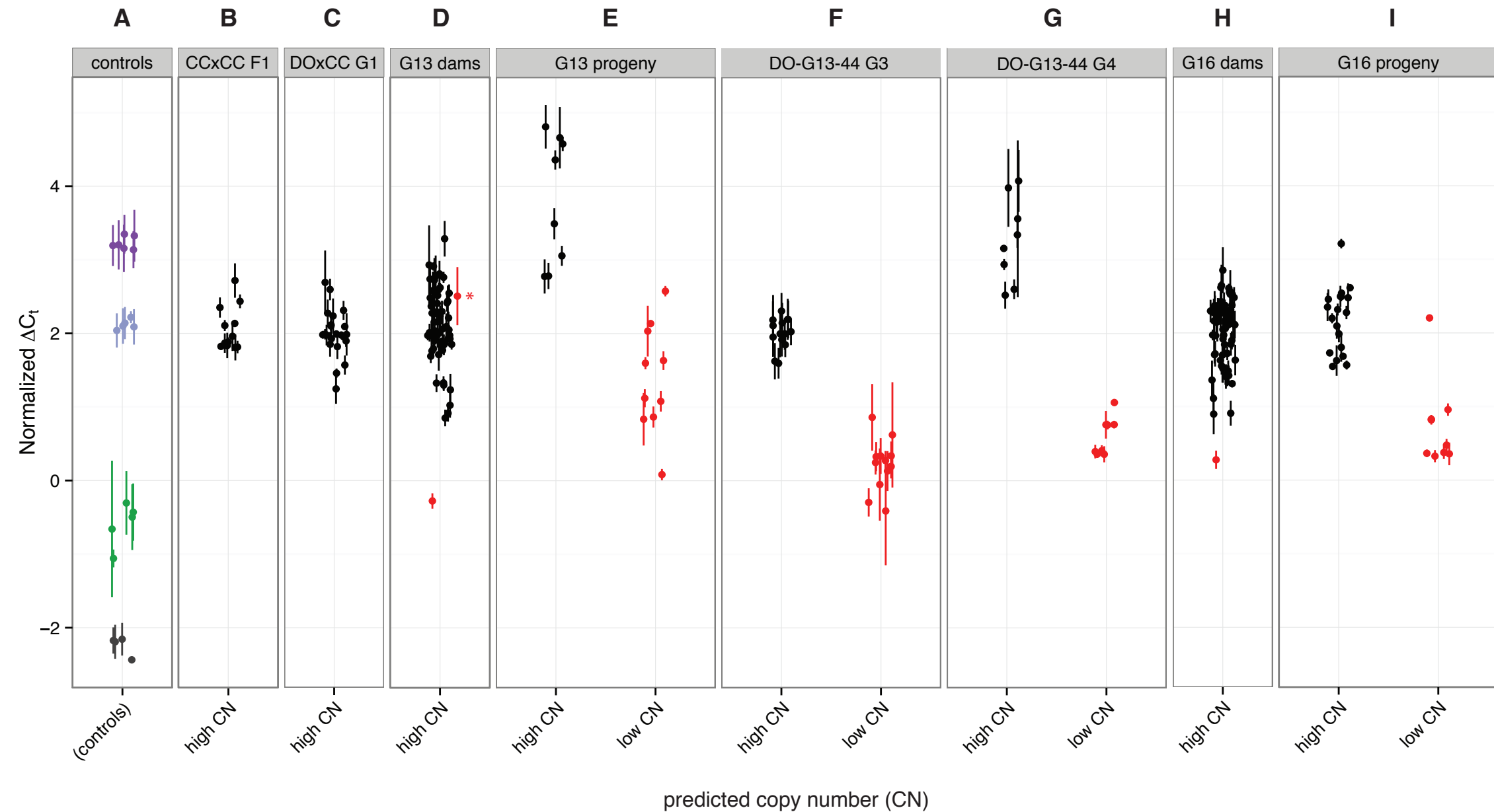

Supplement: S5 Fig — Normalized ΔCt, normalized cycle threshold by TaqMan qPCR assay (see Methods). A) Homozygous calibration samples used for TaqMan assays targeting Cwc22: C57BL/6J (dark grey), haploid copy number 1; CAST/EiJ (green), copy number 2; (WSB/EiJxC57BL/6J)F1 (lavender), copy number ~17; and WSB/EiJ (purple), copy number ~33. In panels B-H, all samples are predicted to be heterozygous for the R2d2 WSB allele based on genotype by PCR at marker Chr2:85.65Mbp. B) F1 hybrids between inbred CC lines used to define the 9.3 Mb candidate interval (see S1 Table). C) G1 hybrids between DO females and CC males used to define R2d candidate interval (see S1 Table). D) Heterozygous DO G13 dams. Outlier sample marked in red is female DO-G13–049, dam of samples in panel G. Sample marked in red and with (*) is female DO-G13–044, the dam of samples in panel H. E) Progeny of DO G13 dams according to predicted copy number (CN), based on TaqMan assay of corresponding G13 dam. Red points are progeny of female DO-G13–049. F) G3 progeny of family DO-G13–44 (see Fig. 3), the offspring of female DO-G13–044, according to predicted CN based on haplotypes linked to R2d. G) G4 progeny in family DO-G13–44, according to predicted CN based on TaqMan assay of corresponding G3 dams. Only low-molecular weight (LMW) DNA was available for samples in panels G and I; note that ΔCt values obtained from LMW DNA are not directly comparable to ΔCt values from high-molecular weight DNA. H) Heterozygous DO-G16 dams. Outlier sample marked in red is DO-G16–107, dam of samples in panel I. I) Progeny of DO-G16 dams according to predicted copy number (CN), based on TaqMan assay of corresponding DO-G16 dam. Red points are progeny of DO-G16–107. (PDF) [file pgen.1004850.s005.pdf]

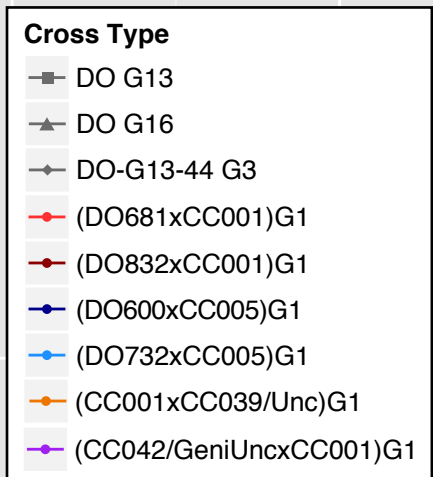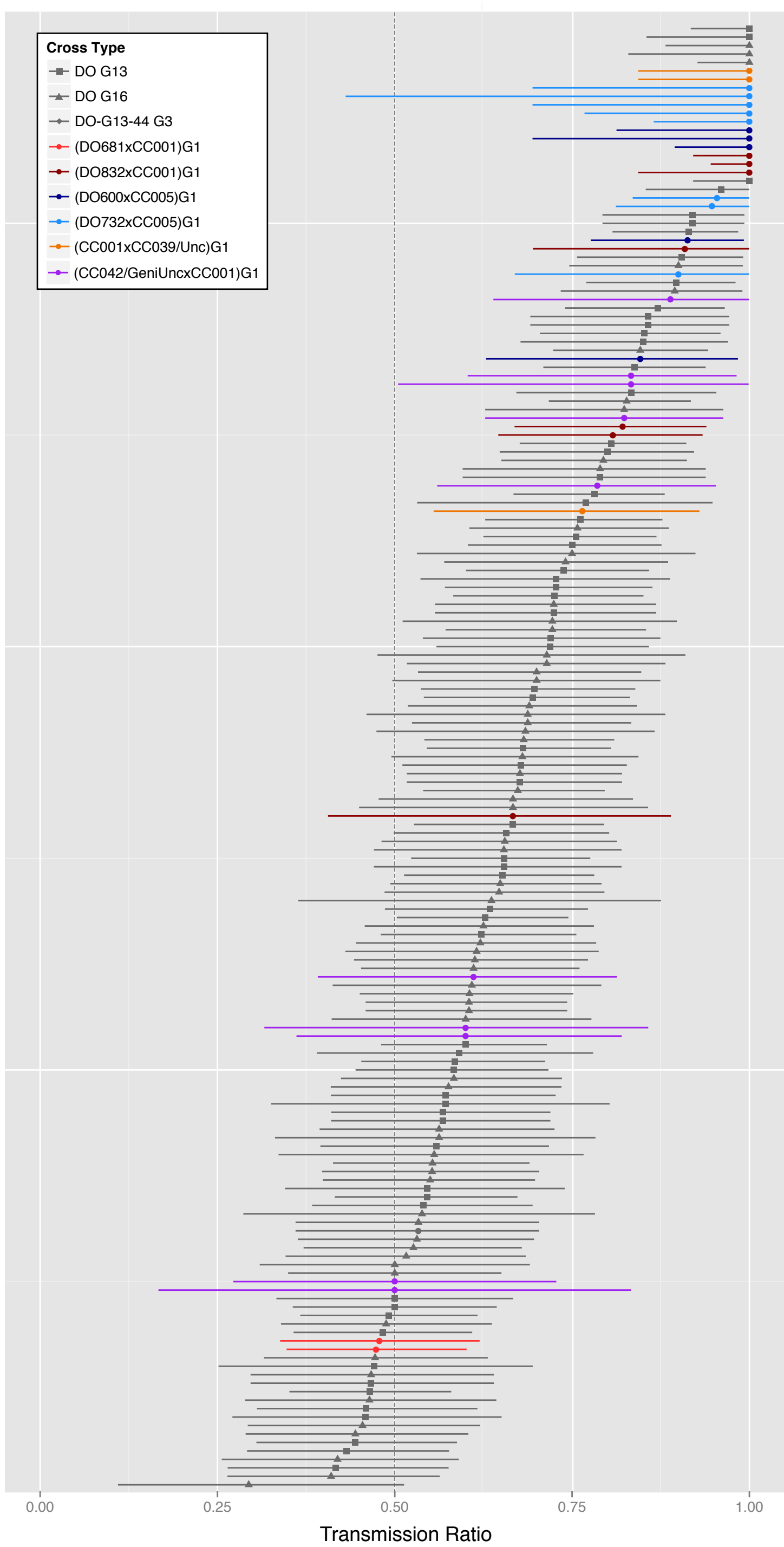

Supplement: S6 Fig — TRs (points) and 95% confidence intervals (lines) for each female from the different types of crosses indicated in the legend. Gray points represent crosses between heterozygous DO females and FVB/NJ males. All other crosses are those that appear in Fig. 1. Females with a mutant R2d2 WSB allele are excluded. Dotted line shows Mendelian expectation of 0.5. (PDF) [file pgen.1004850.s006.pdf]

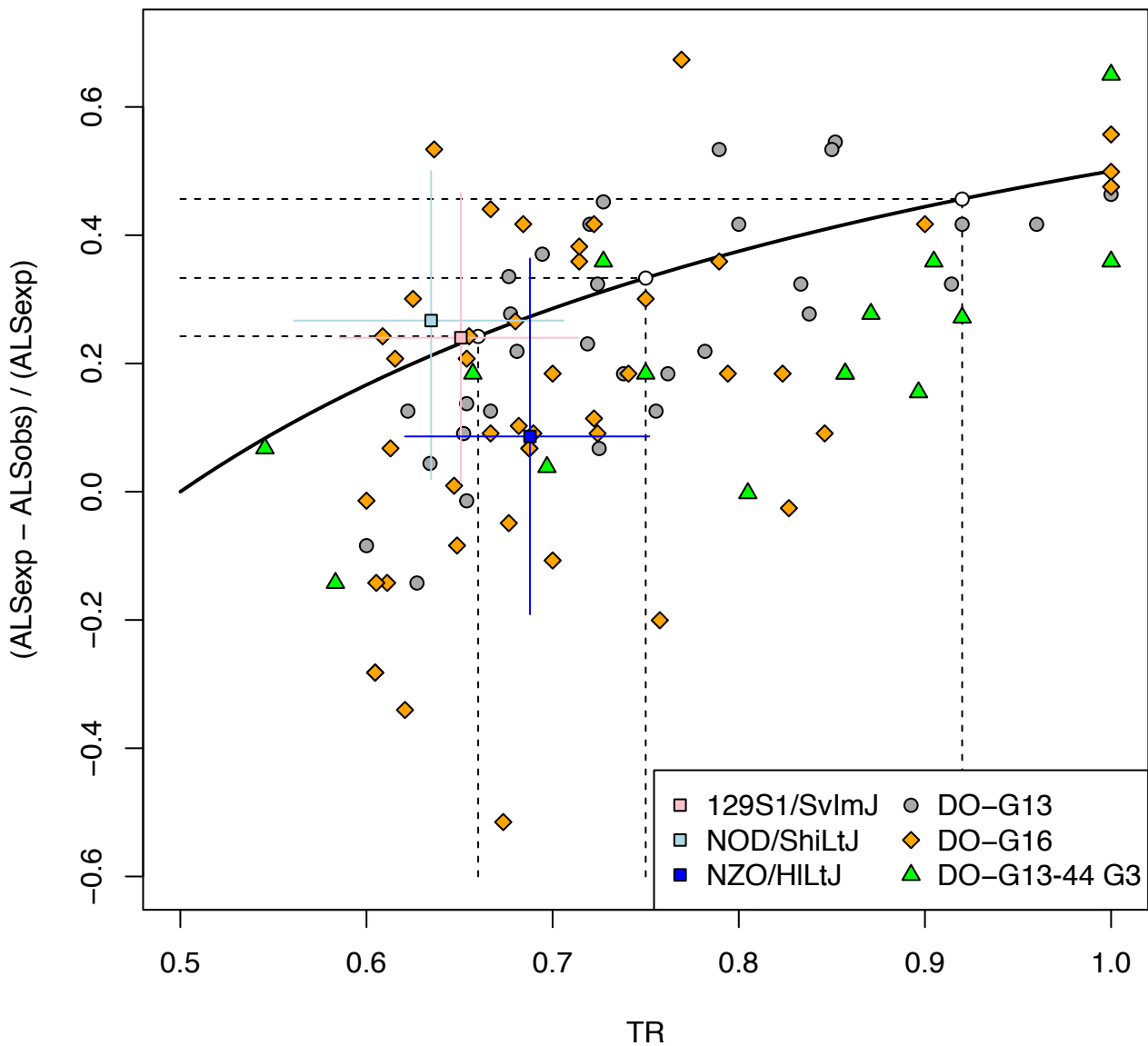

Supplement: S7 Fig — The ratio of observed to expected litter size [(ALS Exp—ALS Obs) / ALS Exp] under a model in which TRD is explained solely by lethality (see Materials and Methods) is shown for TRs between 0.5–1.0 (black line), where expected litter size is 8.4 (the mean litter size of DO females without TRD). Dotted lines show the relationship between lethality and TR for three representative TR values (0.92 represents the threshold used in the text to define DO females with extreme TRD); for each value, the expected ratio and equivalent litter size are shown. Colored squares show aggregate values for three of the test crosses shown in Table 1: light blue = cross 12, (NOD/ShiLtJxWSB/EiJ)F1; pink = cross 13, (129S1/SvImJxWSB/EiJ)F1; dark blue = cross 15, (NZO/HlLtJxWSB/EiJ)F1. Other shapes show values for individual DO females (identified with “**” in S1 Table). Females with a mutant R2d2 WSB allele are excluded. Note that females below the black line have TRs that are too high to be explained solely by lethality given their average litter sizes. (PDF) [file pgen.1004850.s007.pdf]

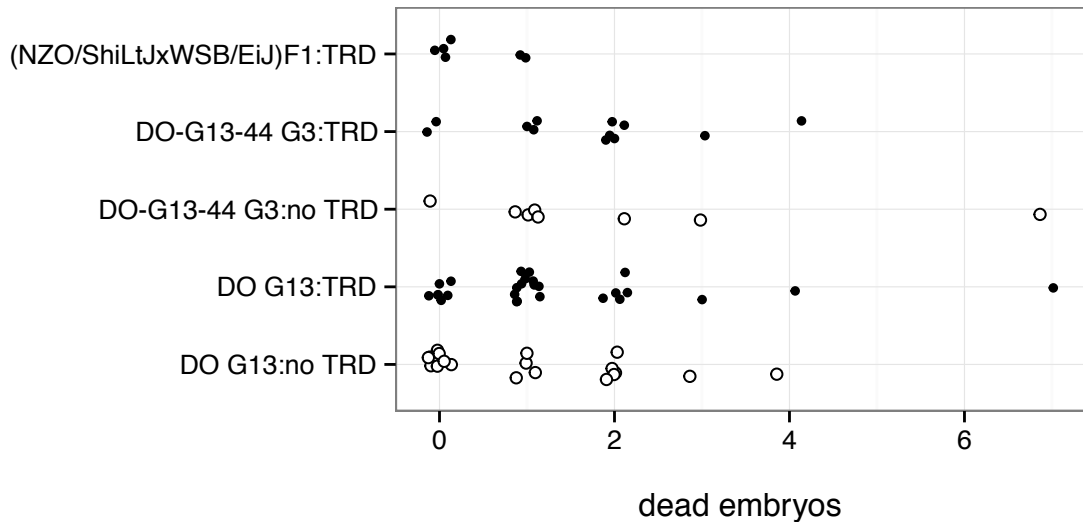

Supplement: S8 Fig — Count of dead embryos per dam at mid-gestation in heterozygous dams. Filled points, dams with TRD; open points, dams with no TRD. Points are jittered to reveal coincident values. (PDF) [file pgen.1004850.s008.pdf]

**A**

ovulation rate

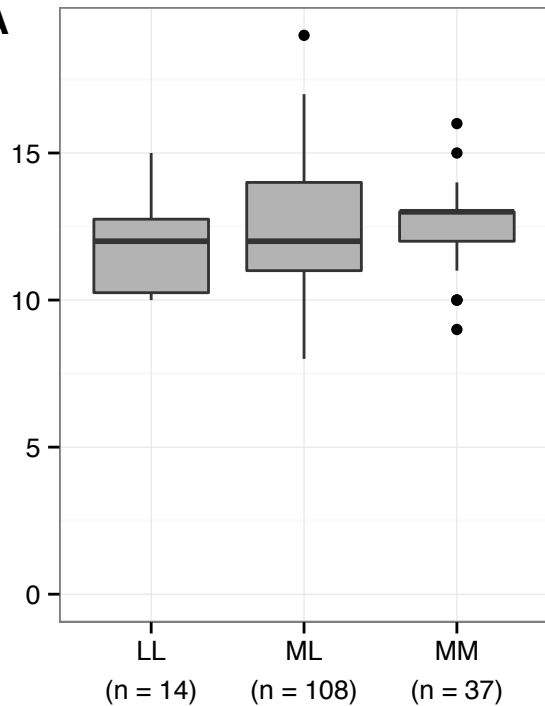**B**

live embryos

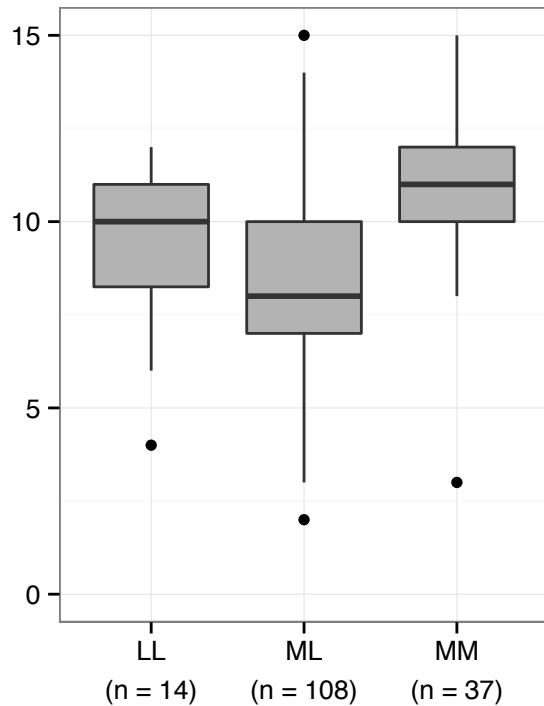

Supplement: S9 Fig — Ovulation rate, in oocytes per dam A), and count of live embryos per dam B), according to genotype at R2d2, assayed by TaqMan. Genotypes are coded as LL = homozygous L6, ML = heterozygous, MM = homozygous M16i. (PDF) [file pgen.1004850.s009.pdf]

Haplotype found in:

A

*R2d2*

*cis*

*trans*

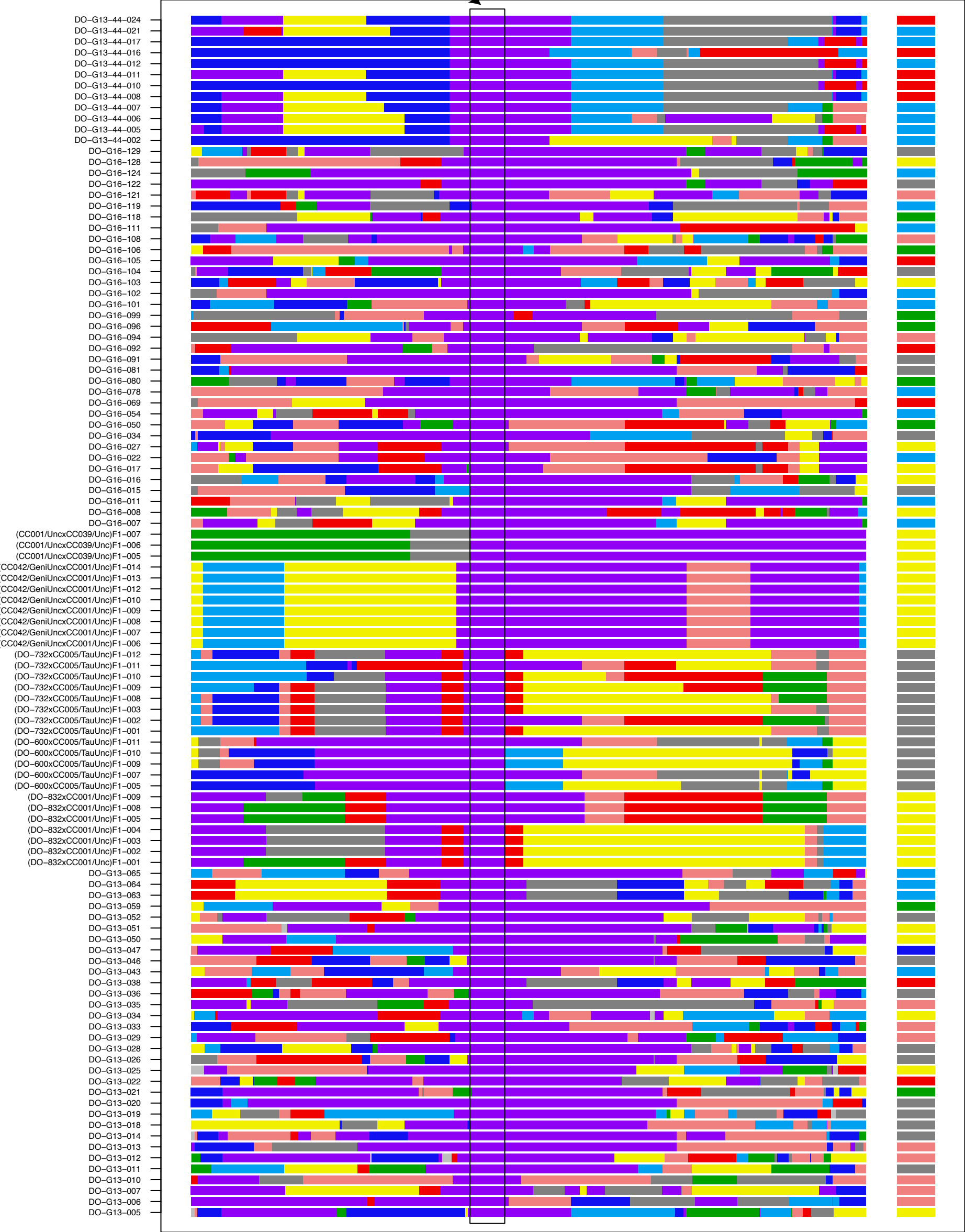

B

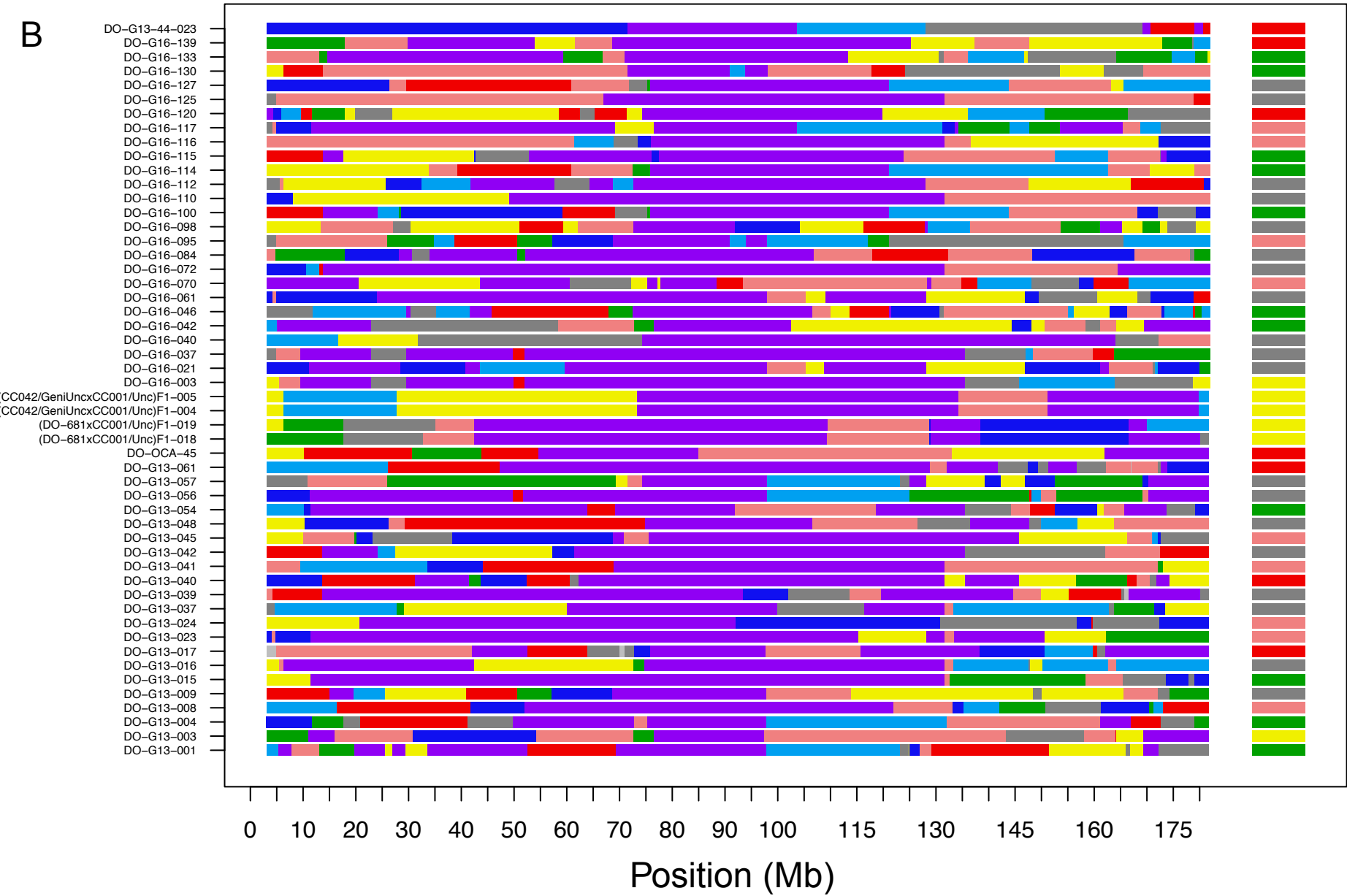

Supplement: S10 Fig — Chr 2 haplotypes in R2d2 heterozygous dams assessed for TR. Shown colored by CC founder strain (see legend in S2 Fig.) are the haplotypes found in cis (left panel) and trans (right panel) to the WSB/EiJ allele in females A) with TRD and B) without TRD. Females with a mutant R2d2 WSB allele are excluded. The black box shows the boundaries of the R2d2 candidate interval. (PDF) [file pgen.1004850.s010.pdf]

DO-G13-049

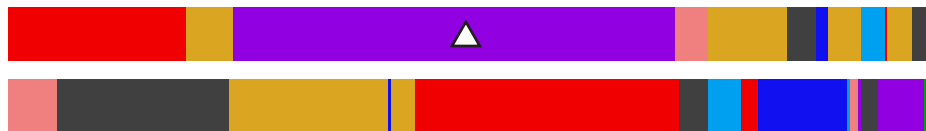

**5**  
( $4.6 \pm 0.1$ )

DO-G16-107

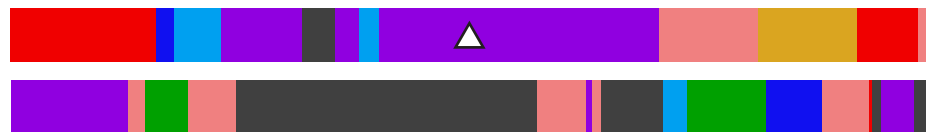

**9**  
( $9.1 \pm 2.0$ )

DO-G13-044

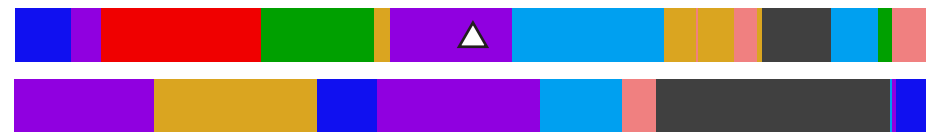

**11**  
( $11.1 \pm 6.8$ )

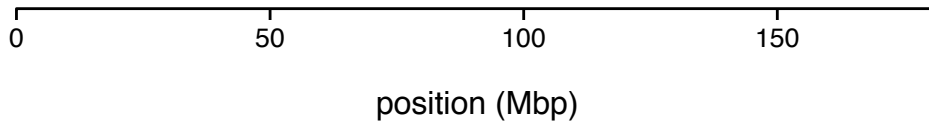

Supplement: S11 Fig — Haplotypes are shown colored by CC founder strain (see legend in S2 Fig.). White Δ indicates location of deletion. Phasing is arbitrary except in DO-G13–044 (G2 dam in family DO-G13–44), whose haplotypes could be phased by manual inspection of offspring genotypes. Copy number at the R2d2 locus for each chromosome (estimated from TaqMan normalized ΔCt values in progeny bearing that chromosome) is indicated at right: first the best estimate of integer copy number, then mean of point estimates across progeny ± 1 standard error. (PDF) [file pgen.1004850.s011.pdf]

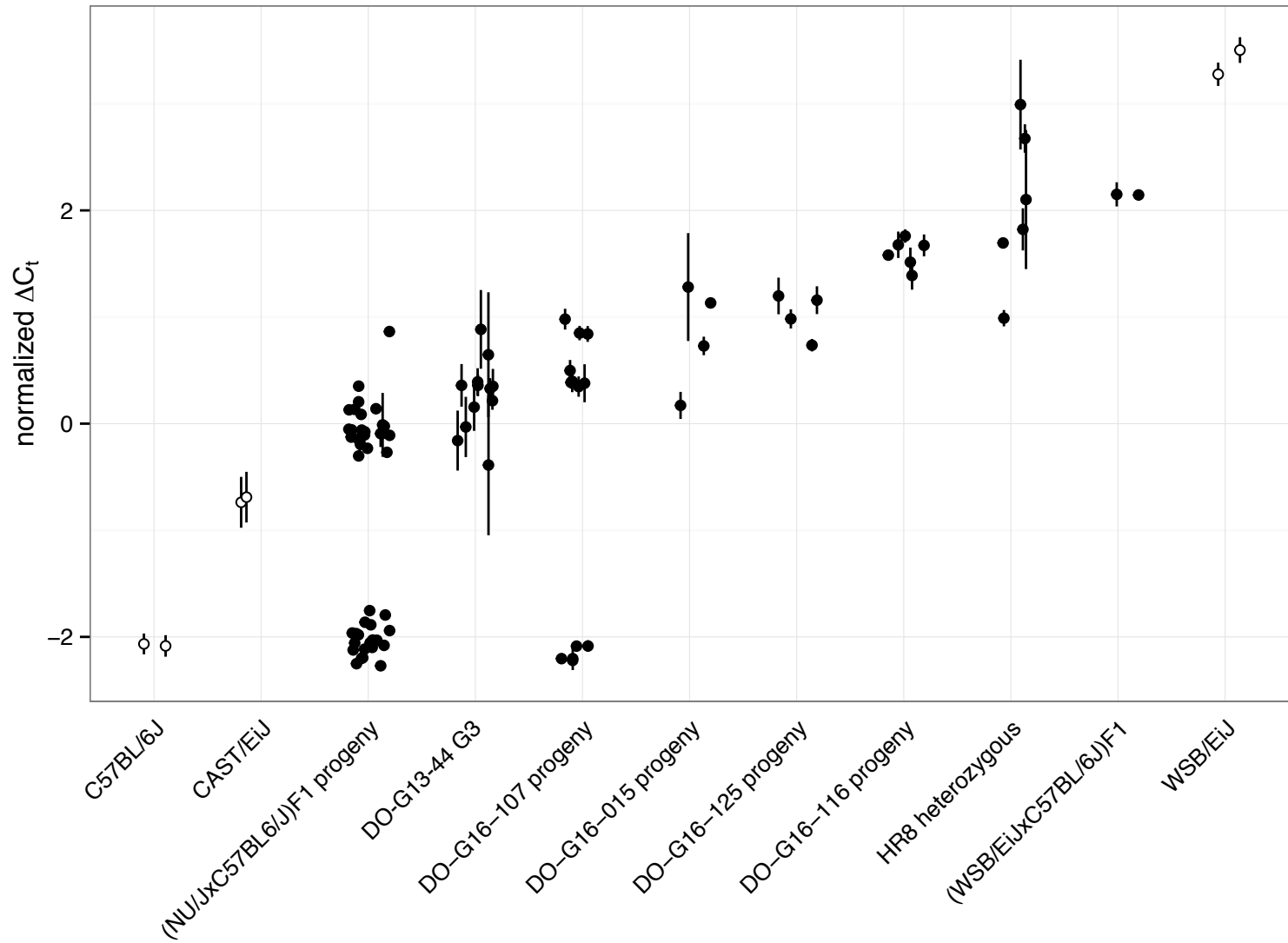

Supplement: S12 Fig — Filled points, heterozygous samples; open points, homozygous control samples. Progeny can be clearly divided into two classes (high normalized ΔCt, NU/J or WSB/EiJ allele; low normalized ΔCt, alternate allele), demonstrating that the TaqMan assay is appropriate for genotyping at R2d2. Progeny of additional DO-G13 and DO-G16 samples suspected to carry low-copy alleles are shown for comparison. (PDF) [file pgen.1004850.s012.pdf]
