# Supplementary material for: A Multi-Megabase Copy Number Gain Causes Maternal Transmission Ratio Distortion on Mouse Chromosome 2
Source: PLoS Genet. 2015 Feb 13;11(2):e1004850. doi: 10.1371/journal.pgen.1004850 (PMC4334553; doi:10.1371/journal.pgen.1004850)
Supplement: S4 Fig — The recombinant haplotypes and sum intensities A) for 34 MDA probes in 58 mice defining the boundaries of copy-number gain in the CAST/EiJ strain, and B) 3 MegaMUGA probes in 74 mice defining the boundaries of copy-number gain in the WSB/EiJ strain. Haplotypes are colored as in the legend in S2 Fig.. C) Distribution of sum-intensity for the three probes in the R2d2 copy number gain region present on MegaMUGA for offspring of a (C57BL/6JxSPRET/EiJ)F1xC57BL/6J (BSB) backcross or a (A/JxSPRET/EiJ)F1xA/J (ASA) backcross is shown in the top right panel, and the sum intensities and recombinant haplotypes in the mice are shown below. Haplotypes are colored by parental strain: SPRET/EiJ (brown), C57BL/6J (black) or A/J (yellow). The high sum intensity, associated with a copy number gain, that is present in ASA-74-B localizes R2d2 distal to the location of the unique copy in the reference sequence (gray dotted line). (PDF) [file pgen.1004850.s004.pdf]

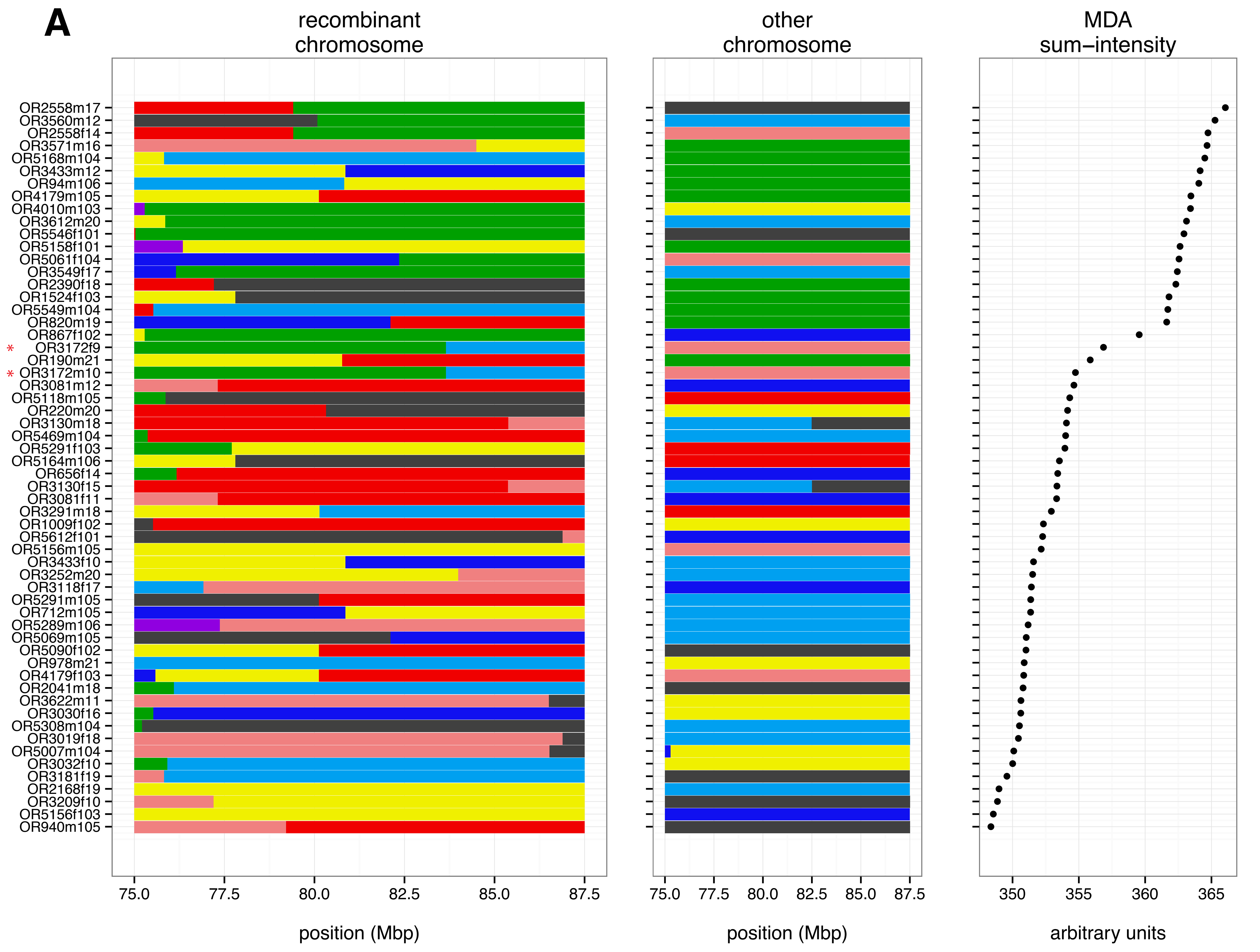
A
recombinant chromosome
other chromosome
MDA sum−intensity
OR2558m17
OR3560m12
OR2558f14
OR3571m16
OR5168m104
OR3433m12
OR94m106
OR4179m105
OR4010m103
OR3612m20
OR5546f101
OR5158f101
OR5061f104
OR3549f17
OR2390f18
OR1524f103
OR5549m104
OR820m19
OR867f102
* OR3172f9
OR190m21
* OR3172m10
OR3081m12
OR5118m105
OR220m20
OR3130m18
OR5469m104
OR5291f103
OR5164m106
OR656f14
OR3130f15
OR3081f11
OR3291m18
OR1009f102
OR5612f101
OR5156m105
OR3433f10
OR3252m20
OR3118f17
OR5291m105
OR712m105
OR5289m106
OR5069m105
OR5090f102
OR978m21
OR4179f103
OR2041m18
OR3622m11
OR3030f16
OR5308m104
OR3019f18
OR5007m104
OR3032f10
OR3181f19
OR2168f19
OR3209f10
OR5156f103
OR940m105
75.0 77.5 80.0 82.5 85.0 87.5
position (Mbp)
75.0 77.5 80.0 82.5 85.0 87.5
position (Mbp)
350 355 360 365
arbitrary units

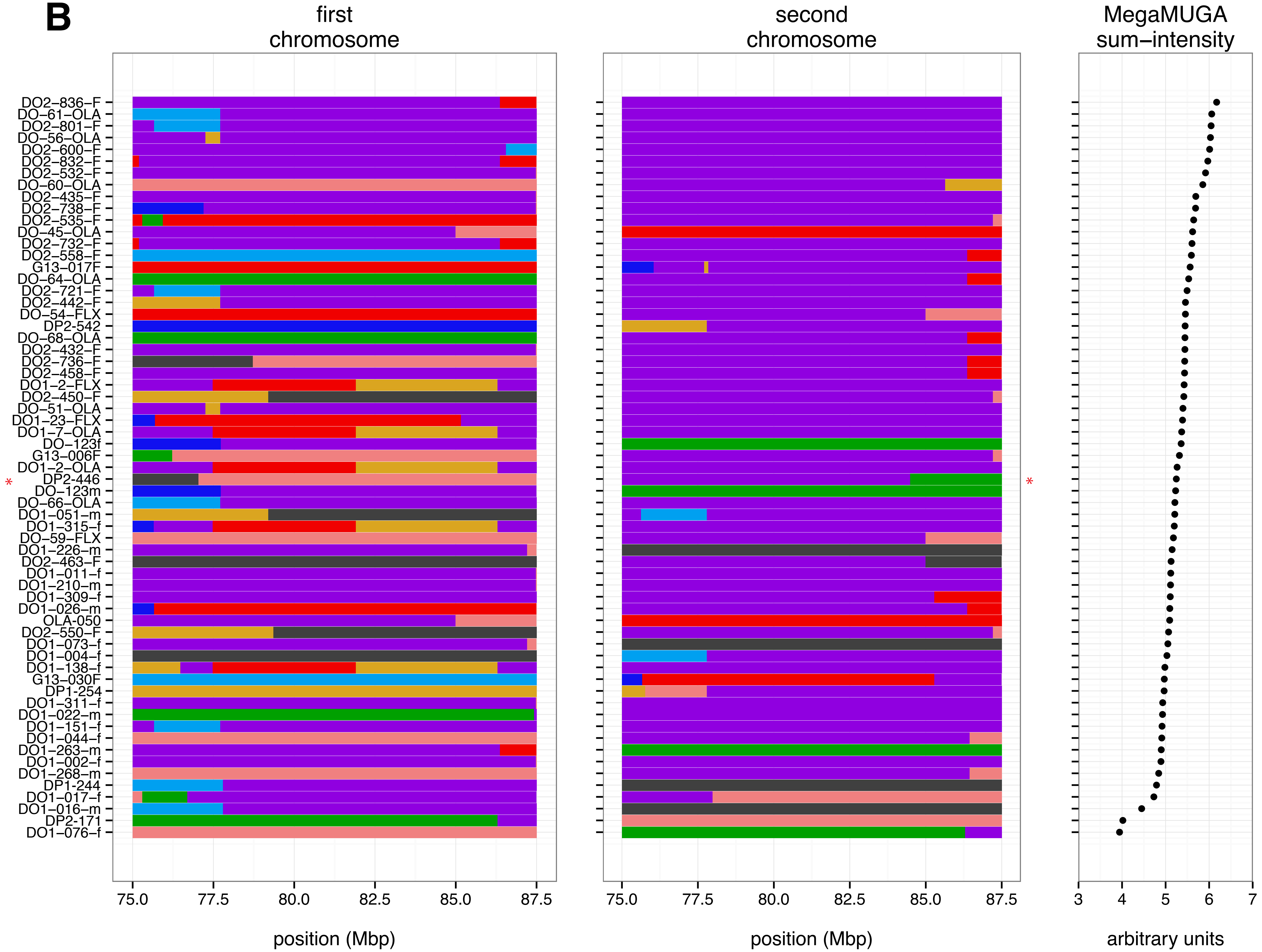
B
first chromosome
second chromosome
MegaMUGA sum−intensity
DO2−836−F
DO−61−OLA
DO2−801−F
DO−56−OLA
DO2−600−F
DO2−832−F
DO2−532−F
DO−60−OLA
DO2−435−F
DO2−738−F
DO2−535−F
DO−45−OLA
DO2−732−F
DO2−558−F
G13−017F
DO−64−OLA
DO2−721−F
DO2−442−F
DO−54−FLX
DP2-542
DO−68−OLA
DO2−432−F
DO2−736−F
DO2−458−F
DO1−2−FLX
DO2−450−F
DO−51−OLA
DO1−23−FLX
DO1−7−OLA
DO−123f
G13−006F
DO1−2−OLA
*
DP2-446
DO−123m
DO−66−OLA
DO1−051−m
DO1−315−f
DO−59−FLX
DO1−226−m
DO2−463−F
DO1−011−f
DO1−210−m
DO1−309−f
DO1−026−m
OLA-050
DO2−550−F
DO1−073−f
DO1−004−f
DO1−138−f
G13−030F
DP1-254
DO1−311−f
DO1−022−m
DO1−151−f
DO1−044−f
DO1−263−m
DO1−002−f
DO1−268−m
DP1-244
DO1−017−f
DO1−016−m
DP2-171
DO1−076−f
75.0
77.5
80.0
82.5
85.0
87.5
position (Mbp)
3
4
5
6
7
arbitrary units

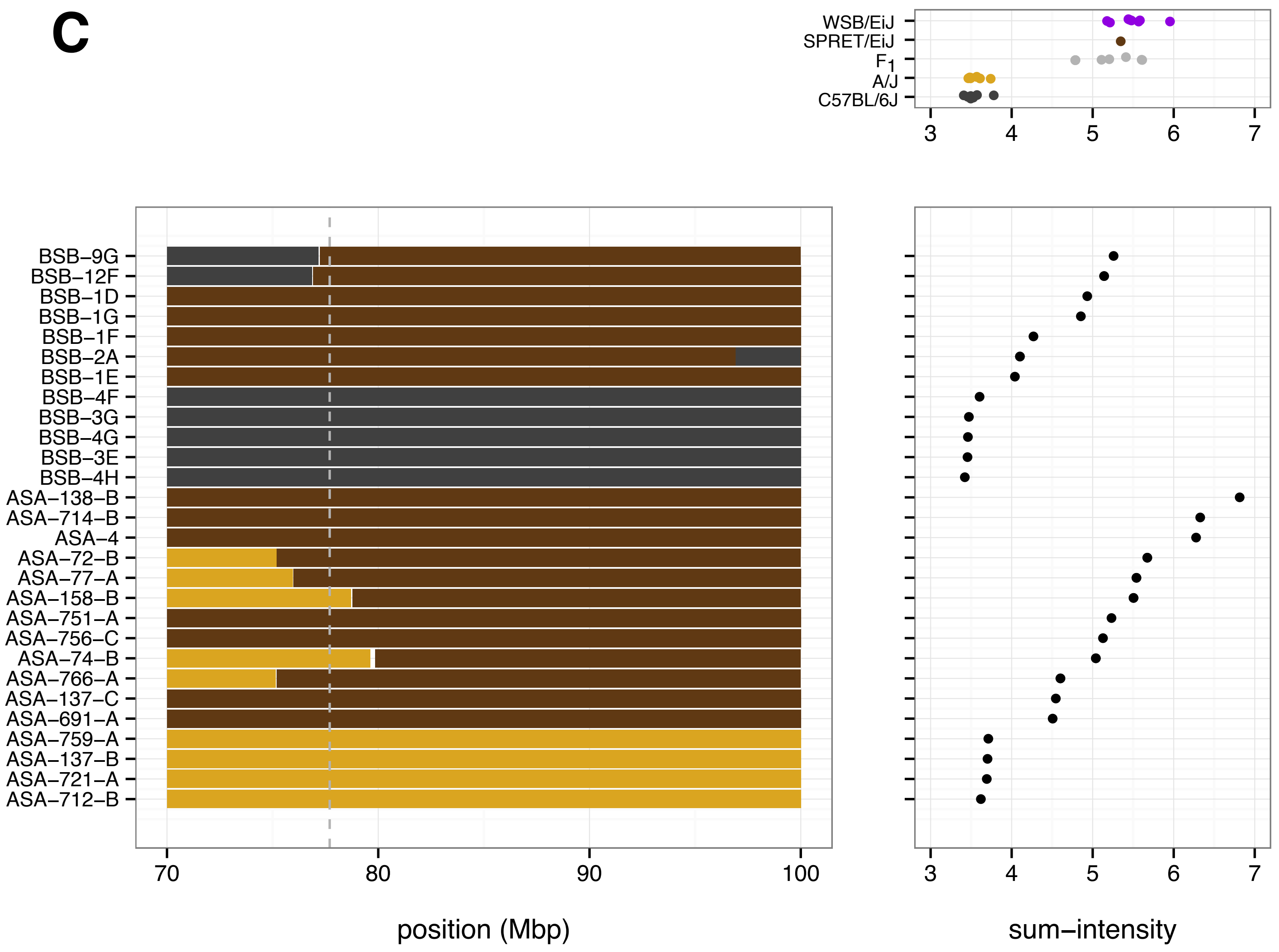
C
WSB/EiJ
SPRET/EiJ
F1
A/J
C57BL/6J
3
4
5
6
7
BSB−9G
BSB−12F
BSB−1D
BSB−1G
BSB−1F
BSB−2A
BSB−1E
BSB−4F
BSB−3G
BSB−4G
BSB−3E
BSB−4H
ASA−138−B
ASA−714−B
ASA−4
ASA−72−B
ASA−77−A
ASA−158−B
ASA−751−A
ASA−756−C
ASA−74−B
ASA−766−A
ASA−137−C
ASA−691−A
ASA−759−A
ASA−137−B
ASA−721−A
ASA−712−B
70
80
90
100
position (Mbp)
3
4
5
6
7
sum−intensity
